# A Peptide from Budding Yeast GAPDH Serves as a Promising Antifungal against Cryptococcus neoformans

**DOI:** 10.1128/spectrum.00826-21

**Published:** 2022-01-12

**Authors:** Yang Zhang, Liyan Zhou, Yan Liu, Xi Zhao, Xianqiang Lian, Jie Zhang, De Zhao, Yujuan Wang, Jin Zhong, Junfeng Wang, Hongli Wang, Linqi Wang, Yu V. Fu

**Affiliations:** a School of Life Sciences, Division of Life Sciences and Medicine, University of Science and Technology of China, Hefei, China; b State Key Laboratory of Microbial Resources, Institute of Microbiology, Chinese Academy of Sciences, Beijing, China; c Savaid Medical School, University of Chinese Academy of Sciences, Beijing, China; d High Magnetic Field Laboratory, Hefei Institutes of Physical Science, Chinese Academy of Sciences, Hefei, China; e Department of Laboratory Medicine, Beijing Obstetrics and Gynecology Hospital, Capital Medical University, Beijing, China; University of Exeter

**Keywords:** antifungal peptide, GAPDH, *Cryptococcus neoformans*, apoptosis

## Abstract

Infection of Cryptococcus neoformans is one of the leading causes of morbidity and mortality, particularly among immunocompromised patients. However, currently available drugs for the treatment of C. neoformans infection are minimal. Here, we report SP1, a peptide derived from glyceraldehyde-3-phosphate dehydrogenase (GAPDH) of Saccharomyces cerevisiae, efficiently kills C. neoformans and Cryptococcus gattii. SP1 causes damages to the capsule. Unlike many antimicrobial peptides, SP1 does not form pores on the cell membrane of C. neoformans. It interacts with membrane ergosterol and enters vacuole possibly through membrane trafficking. C. neoformans treated with SP1 show the apoptotic phenotypes such as imbalance of calcium ion homeostasis, reactive oxygen increment, phosphatidylserine exposure, and nuclear fragmentation. Our data imply that SP1 has the potential to be developed into a treatment option for cryptococcosis.

**IMPORTANCE** Cryptococcus neoformans and Cryptococcus gattii can cause cryptococcosis, which has a high mortality rate. To treat the disease, amphotericin B and fluconazole are often used in clinic. However, amphotericin B has rather high renal toxicity, and tolerance to these drugs are quicky developed. The peptide SP1 derived from baker’s yeast GAPDH shows antifungal function to kill Cryptococcus neoformans and Cryptococcus gattii efficiently with a high specificity, even for the drug-resistant strains. Our data demonstrate that SP1 induces the apoptosis-like death of Cryptococcus neoformans at low concentrations. The finding of this peptide may shed light on a new direction to treat cryptococcosis.

## INTRODUCTION

Cryptococcosis is a life-threatening infection of the central nervous system and lungs caused by the encapsulated yeast species Cryptococcus neoformans and Cryptococcus gattii (1, 2). Globally, cryptococcal meningitis is responsible for 181,100 deaths annually (3) and is exacerbated by the shortage of available treatments, which currently includes amphotericin B (AmB), azoles, and 5-flucytosine (5-Fu) treatments in the clinic (4). Additionally, many studies revealed that an elevated mutation rate in C. neoformans cells increases the development of resistance to these drugs (5). The prevalence of C. neoformans infections is increasing globally and antimicrobial resistance is a significant public health concern (6).

The ubiquitous and relentless clinical challenge of drug resistance has created a pressing need for new agents. Antimicrobial peptides (AMPs) have been presented as a potential solution because of their mechanisms of action (7). Most AMPs are innate immune system components that are produced by a wide variety of organisms, including invertebrates, plants, and animals (8, 9).

Since the first AMP was discovered in the nineteenth century, many additional peptides have emerged that possess antimicrobial activities (10). The anti-C. neoformans peptides found to date exhibit one of two types of antifungal activity (11, 12). The first type of anti-C. neoformans activity is attributed to a membrane-lytic mechanism that directly interferes with the integrity of the fungal cell membrane (13). Peptides with this activity form a transmembrane channel in the membrane via self-aggregation or polymerization, leading to cytoplasm leakage and cell death (13). Examples of this type of AMP include alamethicin and melittin, which are linear cationic α-helical peptides with an amphiphilic surface that can penetrate the cell membrane to exhibit strong membrane-lytic activity (14, 15). The second type of anti-C. neoformans activity is described as intracellular inhibitory activity that is the primary or supportive mechanism for achieving efficient killing (16), as in the antifungal peptide, VG16KRKP (17).

Recent studies reported that AMPs derived from Saccharomyces cerevisiae glyceraldehyde-3-phosphate dehydrogenase (GAPDH) protein can kill competing species during fermentation (18). GAPDH plays a vital role in glycolysis (19). Nevertheless, emerging evidence suggests that GAPDH is a highly versatile protein (20). It has diverse roles across several pathways, including in the regulation of cell death, autophagy, DNA replication, DNA repair, gene expression, RNA export, and membrane fusion (21). It has been shown that AMPs derived from the C-terminal fragment of GAPDH from S. cerevisiae can inhibit the growth of Kluyveromyces marxianus and Kluyveromyces thermotolerans during alcoholic fermentation performed with mixed cultures (22). Moreover, GAPDH from yellowfin tuna demonstrated potent antimicrobial activity against Gram-positive bacteria, such as Bacillus subtilis, Micrococcus luteus, and Streptococcus iniae (23, 24). Furthermore, it has been shown that the N-terminal fragment of the human GAPDH protein exhibits anti-Candida albicans activity (25). The detailed molecular mechanisms underlying the antifungal effect of these peptides remain unknown.

In this study, we identified a peptide, SP1, derived from the N-terminal of the GAPDH protein from S. cerevisiae that possesses antifungal potential. SP1 had specific antimicrobial activity against the opportunistic fungal pathogens C. neoformans and C. gattii. Our data suggest that SP1 can damage the capsule of C. neoformans, leading to apoptosis-like cell death, potentially by disrupting calcium homeostasis, mitochondrial dysfunction, and reactive oxygen species (ROS) accumulation.

## RESULTS

### Anti-C. neoformans activity of SP1.

SP1 is a peptide derived from the N-terminal of the S. cerevisiae GAPDH protein, and its amino acid sequence is IRIAINGFGRIGRLVLRLALQRKDIEVVA. Because S. cerevisiae produces peptides from GAPDH to kill other yeast competitors during wine fermentation (22), we were interested in whether SP1 may have the ability to kill other yeast species, such as pathogenic yeasts.

To test whether SP1 has antimicrobial activity against pathogenic yeasts, SP1 was chemically synthesized based on the amino acid sequence indicated above. The MIC of the peptide was determined using a standardized micro-broth dilution method with a panel of pathogenic yeast strains (C. albicans, A. fumigatus, C. neoformans, and C. gattii) and bacterial strains (Escherichia coli, Micrococcus luteus, Staphylococcus aureus, and Pseudomonas aeruginosa). AmB and melittin were used as positive controls based on their demonstrated effectiveness against fungi. SP1 had no inhibitory activity against the above-mentioned bacteria (Table S1 in the supplemental material), and A. fumigatus was also insensitive to SP1. C. albicans was mildly sensitive to SP1. However, C. neoformans and C. gattii were highly sensitive to SP1 (Table 1). The MIC of SP1 for C. neoformans strains (JEC21 strain, clinical isolate W82 strain, R272 strain) and C. gattii strains (clinical isolate W1011 strain) was 4 μM (Table 1), and the MIC of SP1 for the C. neoformans H99 strain, clinical isolate W83 strain, and C. gattii clinical isolate W1010 strain was 8 μM (Table 1). For the C. neoformans clinical isolate WM179 strain and the C. gattii clinical isolate W1012 and W2520 strains, the MIC of SP1 was 32 μM (see Table S2 in the supplemental material for strain typing information).

**TABLE 1:** The MICs of SP1, Melittin, AmB, and FLC against different fungi^*a*^

Strain	MIC			
	SP1	Melittin	AMB	FLC
Cryptococcus neoformans				
JEC21	4 μM(13 μg/mL)	NA	0.4 µM (0.37 μg/mL)	6.5 µM (2 μg/mL)
H99	8 μM (26 μg/mL)	2.5 µM (7.12 μg/mL)	1.1 µM (1 μg/mL)	6.5 µM (2 μg/mL)
W82	4 μM (13 μg/mL)	NA	NA	NA
W83	8 μM (26 μg/mL)	NA	NA	NA
R272	4 μM(13 μg/mL)	NA	NA	NA
WM179	32 μM (104 μg/mL)	NA	NA	NA
*fzc51Δ*	8 μM (26 μg/mL)	NA	>2.2 µM (2 μg/mL)	6.5 µM (2 μg/mL)
*atf1Δ*	8 μM (26 μg/mL)	NA	>2.2 µM (2 μg/mL)	6.5 µM (2 μg/mL)
*fzc9Δ*	8 μM (26 μg/mL)	NA	1.1 µM (1 μg/mL)	>26 µM (8 μg/mL)
*gat5Δ*	8 μM (26 μg/mL)	NA	1.1 µM (1 μg/mL)	>26 µM (8 μg/mL)
*liv4Δ*	8 μM (26 μg/mL)	NA	1.1 µM (1 μg/mL)	>26 µM (8 μg/mL)
*erg4Δ*	>128 μM (416 μg/mL)	NA	NA	NA
Cryptococcus gattii				
W1011	4 μM (13 μg/mL)	NA	NA	NA
W1010	8 μM (26 μg/mL)	NA	NA	NA
W1012	32 μM (104 μg/mL)	NA	NA	NA
W2520	32 μM (104 μg/mL)	NA	NA	NA
Candida albicans				
	128 μM (416 μg/mL)	NA	0.4 µM (0.37 μg/mL)	NA
Saccharomyces cerevisiae				
	>128 μM (416 μg/mL)	NA	NA	NA
Aspergillus fumigatus				
	>128 μM (416 μg/mL)	NA	0.4 µM (0.37 μg/mL)	NA

a The MIC was determined from three independent experiments performed in triplicate. *C. albicans, S. cerevisiae, A. fumigatus*, Cryptococcus neoformans strains JEC21 and H99 are laboratory stocks. Cryptococcus neoformans strains W82, W83, R272, WM179, and Cryptococcus gattii strains W1011, W1010, W1012, W2520 are clinical isolates. All the FLC and AmB resistant strains are derived from H99, and the *erg4Δ* mutant is derived from H99. AmB, amphotericin; FLC, fluconazole; NA, not available.

To determine whether the SP1 homologous peptide derived from GAPDH of other yeast species has similar anti-cryptococcal effects, we chemically synthesized SP1 homologous peptides derived from C. neoformans (66% sequence identity with SP1), C. albicans (78% sequence identity with SP1), and A. fumigatus (66% sequence identity with SP1), respectively (Fig. S1 in the supplemental material). Of these, only SP1 from GAPDH of S. cerevisiae exhibits the killing ability on C. neoformans and C. gattii (Table S3 in the supplemental material).

To test whether SP1 can inhibit the growth of drug-resistant C. neoformans strains, three fluconazole-(FLC) resistant strains (*fzc9Δ, gat5Δ, liv4Δ*) and two AmB-resistant strains (*fzc51Δ, atf1Δ*) were used to assess the MIC of SP1. As shown in Table 1, the MIC of SP1 for all drug-resistant strains was 8 μM (Table 1). Because SP1 had growth inhibition activity against FLC-resistant and AmB-resistant strains, we hypothesized that it may have the potential to treat infections caused by drug-resistant strains. Interestingly, the *erg4Δ* mutant showed resistance to SP1. *ERG4* encodes a sterol reductase that catalyzes the final step of ergosterol biosynthesis in C. neoformans, and it has been reported that *erg4Δ* strains produce lower levels of ergosterol than wild-type strains of S. cerevisiae (26, 27). Thus, the inhibitory activity of SP1 could be in part due to the level of ergosterol at the cell membrane.

**FIG 1:**
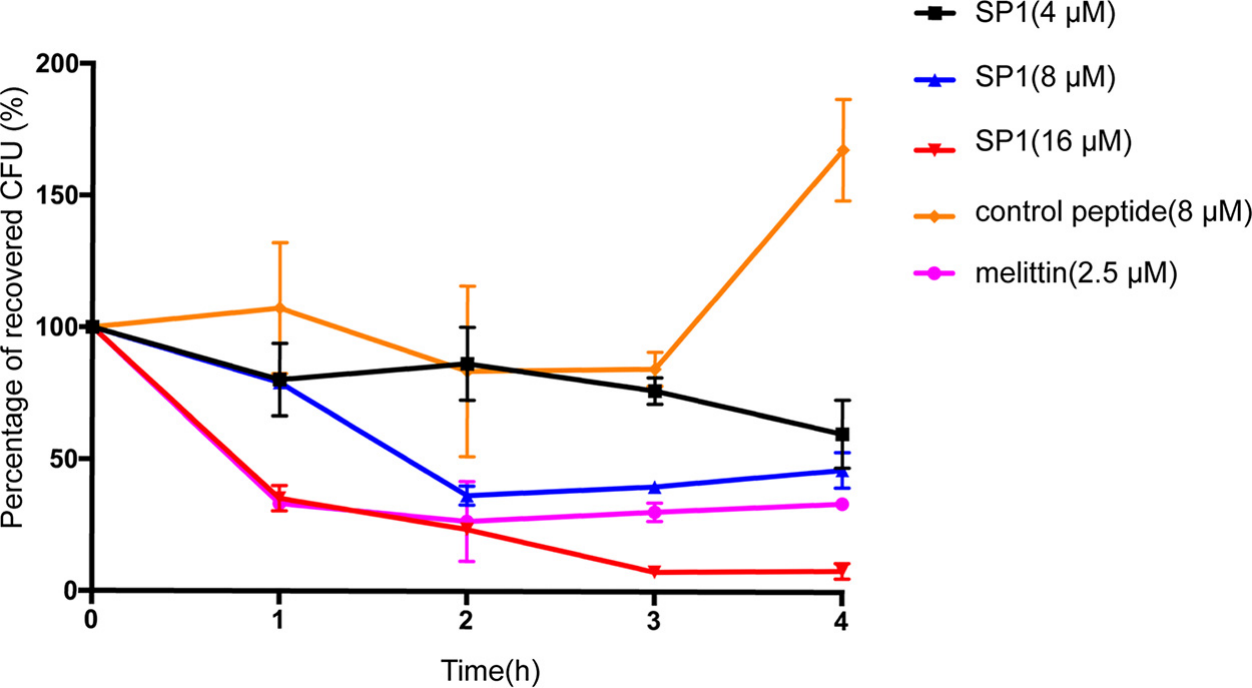
Killing kinetics of SP1. Cryptococcus neoformans H99 killing kinetics upon treatment with SP1 (4 μM,8 μM, or 16 μM), melittin (2.5 μM), or the control peptide (8 μM). The results are presented for three independent experiments.

Next, we tested the killing kinetics of SP1. Melittin was chosen as a positive control for these experiments. Melittin is a small linear cytolytic peptide composed of 26 amino acids that forms pores in the cell membrane, leading to cell death (28). As shown in Fig. 1, 8 μM (MIC) of SP1 killed ∼50% of C. neoformans H99 cells over 4 h, and 2.5 μM (MIC) of melittin killed ∼70% of cells. When the concentration of SP1 reached 16 μM, SP1 killed nearly 90% of the cells during the 4 h of incubation (Fig. 1).

### Characterization of the SP1 secondary structure.

**FIG 2:**
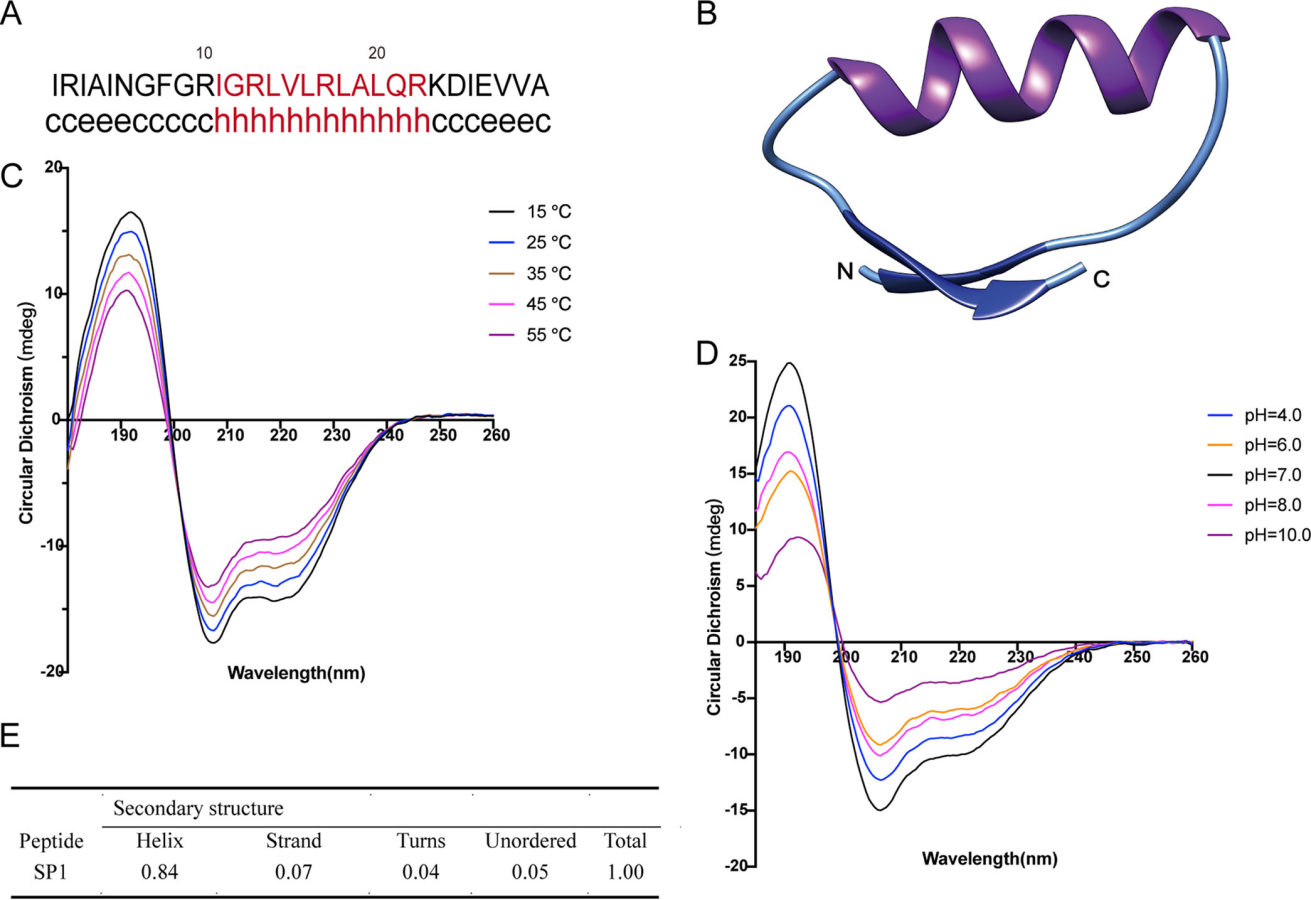
Secondary structure and structural modeling of SP1. (A) The prediction of the secondary structure was performed by the GOR program. The letters c, e, and h represent the secondary structures of a random coil, extended strand, and alpha-helix (red), respectively. (B) The predicted three-dimensional structure of SP1. (C) CD spectrum of SP1 in 50% trifluoroethyl alcohol (TFEA) at different temperatures as indicated. (D) CD spectrum of SP1 in 50% TFEA under various pH values at 37°C. (E) The contents of the secondary structures of SP1 in 50% TFEA at 37°C and pH = 7.0.

The mechanism of AMP antifungal activity is largely attributed to the special structures of AMPs (29, 30). To better understand the anti-C. neoformans mechanisms of SP1, we predicted its secondary structure. The program GOR in Expasy (http://pbil.ibcp.fr/npsa) was used to predict the secondary structure of SP1 (31). The results showed that the central region (amino acids 11 to 22) of SP1 is highly basic and likely to form an α-helix (Fig. 2A). Furthermore, there were two potential small extended sheets at both the N-terminus and C-terminus of SP1 (Fig. 2A).

Because SP1 is derived from GAPDH, we used the crystal structure of GAPDH, which has ∼73% sequence identity with SP1 at its N-terminal (PDB accession no. 2i5o.1.A) for homolog modeling (31). The modeled structure of SP1 had three specific structural signatures: an α-helix strand that spanned the sequence 10RIGRLVLALALQ20, and two β-sheets, 2RIA4 and 26EVVA29, separated by two random coil strands (Fig. 2B).

To further confirm the secondary structure of SP1, the conformational preferences of SP1 were screened by circular dichroism (CD) spectroscopy. The CD spectra of SP1 displayed negative double peaks at 205 nm and 220 nm and a positive peak at 190 nm under different temperatures (Fig. 2C). These spectra are well-known characteristics of an α-helix (32), suggesting that the structure of SP1 is quite stable under different temperatures. The pH stability of SP1 was also determined by CD spectroscopy. Under conditions of pH 4.0 to 10.0 at 37°C, SP1 contained one α-helix structure (Fig. 2D). The secondary structure of SP1 at 37°C and pH 7.0 was calculated from the CD data shown in Fig. 2D. The structure of SP1 was strongly dominated by an α-helix in 50% TFEA solution (Fig. 2E).

### The permeabilization ability of SP1 on different liposomes.

SP1 folds to form an ordered structure in TFEA solution. It has been reported that the amphiphilic nature of TFEA is highly capable of mimicking a cell membrane environment (33). Accordingly, we hypothesized that, similar to several antimicrobial peptides (29, 30), SP1 might interact with the membrane of fungi.

**FIG 3:**
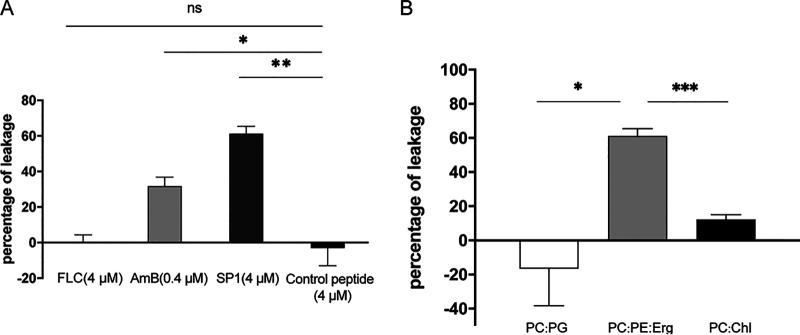
Membrane permeabilization ability of SP1. (A) Bar plot showing the percentage of calcein leakage from POPC/POPE/Erg (5:4:3) vesicles upon treatment with 0.4 μM AmB, 4 μM SP1, 4 μM fluconazole (FLC), or 4 μM control peptide. (**P < 0.01, *P < 0.05). (B) Bar plot showing the percentage of calcein leakage from Phosphatidyl choline: Phosphatidyl glycerol (POPC:POPG), POPC/POPE/Erg (5:4:3), and POPC/40% cholesterol (PC:Chl) vesicles upon treatment with 4 μM SP1. POPC represents 1-palmitoyl-2-oleoyl-sn-glycero-3-phosphoethanolamine, POPE represents 1-palmitoyl-2-linoleoyl-sn-glycero-3-phosphatidylcholine, POPG represents 1-palmitoyl-2-oleoyl-sn-glycero-3-phospho-(1′-rac-glycerol), they all are the main components of the phospholipid bilayer. Erg represents Ergosterol. Error bars represent the standard deviation of three independent experiments. (*P < 0.05, ***P < 0.001).

To test this hypothesis, we investigated the interaction between SP1 and membrane structures using liposome experiments. Calcein is a fluorenone that is self-quenching at high concentrations. When calcein leaks from liposomes, the solution develops an orange color due to the reduction in calcein concentrations (34). Lipid vesicles containing POPC/POPE/ergosterol (Erg) that mimic the membrane of fungi were prepared. SP1 was added to liposomes containing calcein, and the membrane permeability was assessed by monitoring the fluorescence recovery. We added AmB, which is known to cause cell membrane permeability, to POPC/POPE/Erg lipid vesicles as a positive control. FLC and the control peptide were used as negative controls (35). As expected, the assay results showed that FLC and the control peptide did not induce calcein leakage. In contrast, AmB and SP1 each induced varying degrees of calcein leakage (Fig. 3A).

A similar experiment was performed using different vesicle types. For the lipid vesicles of POPC/POPE/Erg, the result revealed a 60% calcein leakage upon the addition of SP1, suggesting a potential interaction between SP1 and the membrane (Fig. 3B). Contrastingly, SP1 caused did not cause calcein leakage from the vesicles composed of phosphatidyl choline (POPC) and phosphatidyl glycerol (POPG), which mimic the membrane of bacteria (Fig. 3B). Moreover, vesicles composed of POPC with 40% cholesterol, which mimics a mammalian cell membrane, demonstrated a negligible amount (10%) of calcein leakage following treatment with the same concentration of SP1.

**FIG 4:**
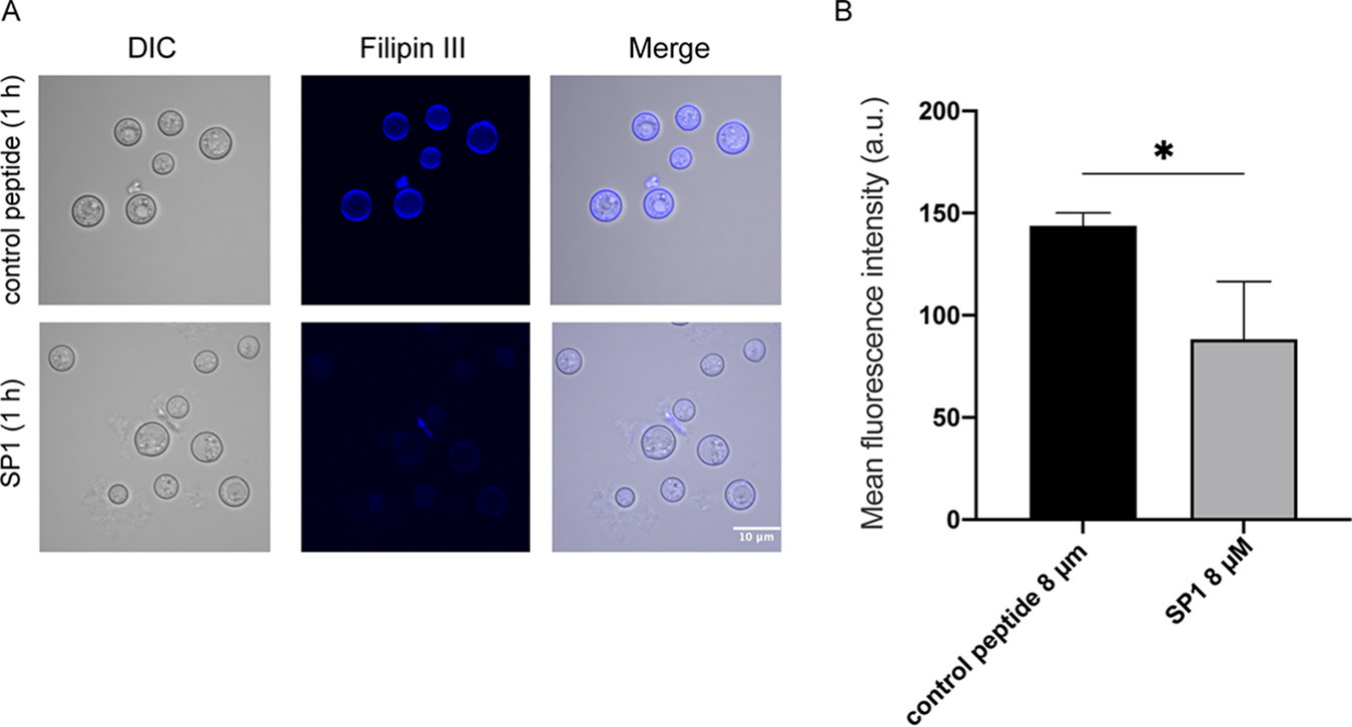
SP1 interacts with the ergosterol in cell membranes. (A) Confocal fluorescence microscopy of the filipin III stained C. neoformans cells. The C. neoformans (H99) cells were treated with 8 μM SP1 or 8 μM control peptide for 1 h before acquiring the images. (B) Mean fluorescence intensity of filipin III stained C. neoformans (H99) cells treated with 8 μM SP1 or 8 μM control peptide for 1 h. The fluorescence intensity was calculated by Image J as described in Methods and Materials; error bars represent the standard deviation of three experiments (*P < 0.05).

As described above, SP1 induced calcein leakage only from lipid vesicles containing POPC/POPE/Erg. Conversely, the *erg4Δ* mutant that contains less ergosterol in the cell membrane of wild type showed resistance to SP1 (Table 1); therefore, we speculated that SP1 might interact with ergosterol in the cell membrane. Filipin III was used to fluorescently mark ergosterol within the cell membrane of SP1- or control peptide-treated cells (36). After a 1 h of incubation, cells treated with SP1 displayed lower fluorescence intensity than cells incubated with the control peptide (Fig. 4A). The quantitative analysis showed that filipin III-stained ergosterol in the cell membrane decreased by ∼40% following 1h SP1 treatment (Fig. 4B). Because the reduced fluorescence intensity occurred within the 1-h incubation period, it is unlikely that SP1 changed the amount of ergosterol in the cell membrane by affecting ergosterol synthesis. The decrease in filipin III staining could have been caused by competition between filipin III and SP1 for ergosterol. In addition, we treated the cells with natamycin or AmB, which are well-known fungicides that specifically bind to ergosterol in the cell membrane (37). For both natamycin and AmB, a decreased level of filipin III signal was observed 1 h after the treatment (Fig. S2 in the supplemental material).

### SP1 does not form pores on the cell membrane.

**FIG 5:**
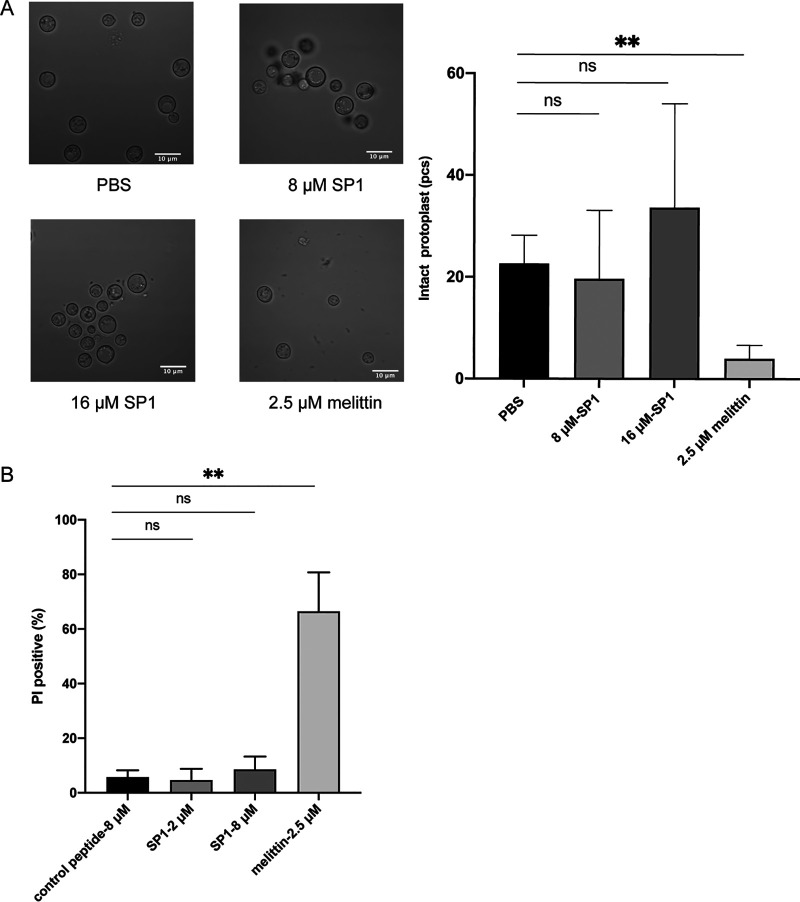
SP1 does not form pores on the cell membrane. (A) The images of C. neoformans protoplasts treated with control peptide, SP1 or melittin. Images were taken by confocal laser-scanning microscopy after 2 h treatment. The right panel shows the statistical results of intact protoplast counting under different treatments, error bars represent the standard deviation of three experiments. (**P < 0.01). (B) Quantification of PI positive C. neoformans protoplasts after SP1 treatment. The H99 protoplast was treated with control peptide, SP1, and melittin for 1 h. Flow cytometry analysis of PI staining was performed as described in “Methods and Materials.” Each experiment used around 30,000 protoplasts per treatment were detected by flow cytometry in three independent experiments, error bars represent the standard deviation of three experiments (**P < 0.01, ****P < 0.0001).

Many antimicrobial peptides form pores in cell membranes to kill cells (38). In accordance with the secondary structure of SP1 and based on the observations from the membrane permeabilization experiments, it is possible that SP1 could form pores on the membrane via interaction with ergosterol. To test this hypothesis, C. neoformans protoplasts were adopted for cell rupture experiments. Because the cell walls were digested with enzymes, the unprotected protoplasts could be easily ruptured following peptide-induced pore formation within the cell membrane. Moreover, without the structural support of a cell wall, cell rupture could be clearly observed under the microscope. The C. neoformans protoplasts were treated with SP1 for 2 h and viewed with a microscope; melittin was used as a positive control. As shown in Fig. 5A, nearly 80% of protoplasts were ruptured after the treatment of 2.5 μM melittin for 2 h. However, the protoplasts maintained their round shape after SP1 treatment, even following treatment with 16 μM SP1 for 2 h. These observations imply that, unlike melittin, SP1 likely does not form pores in cell membranes.

To obtain additional independent evidence for membrane integrity, we treated the C. neoformans protoplasts with SP1 or melittin for 1 h and then treated the cells with propidium iodide (PI). PI is non-membrane-permeable fluorescent intercalating agent used to stain nucleic acids; hence, PI can only enter cells with compromised membranes. Following treatment with 2.5 μM melittin, nearly 70% of cells showed positive PI staining (Fig. 5B). Conversely, cells that were treated with 8 μM SP1 showed no significant differences in PI-positive staining compared with the control group (Fig. 5B). These observations further demonstrate that SP1 is unlikely to form pores on the membrane.

### SP1 damages the capsule of C. neoformans.

**FIG 6:**
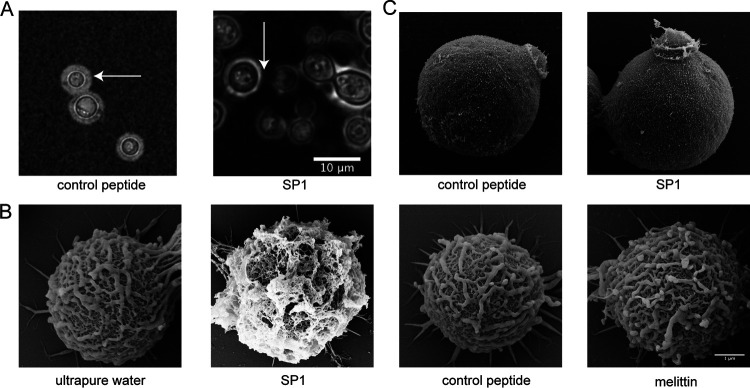
SP1 damages the capsule of C. neoformans. (A) Confocal microscopy of C. neoformans cells treated with SP1 or control peptide. The cells were stained by India ink. The white arrow indicates the capsule. (B) Scanning electron microscopy of C. neoformans cells treated with ultrapure water, 8 μM SP1, 8 μM control peptide, or 2.5 μM melittin, respectively. (C) Scanning electron microscopy of C. neoformans *cap59△* mutant cells treated with 8 μM control peptide or 8 μM SP1.

Although it is unlikely that SP1 forms pores on the cell membrane, interestingly, we observed that the polysaccharide capsule of C. neoformans H99 cells was severely damaged after SP1 treatment for 1 h at a dose of 8 μM (Fig. 6A). To better understand the cell morphology changes that occur following treatment with SP1, we used scanning electron microscopy (SEM) to image the C. neoformans cells. In the SEM images, the capsule appears as short fibrils radiating from the cell surface. After treatment with 8 μM SP1 for 30 min, the short radiating fibrils were severely deformed, and the treatment caused a compact pasted appearance (Fig. 6B). However, no obvious changes in the cellular morphology were observed in the cells treated with water, control peptide, or melittin (Fig. 6B). To examine the capsule damage caused by SP1 and to determine whether these effects happened before or after cell death, we employed PI-stained H99 cells that were treated with 8 μM and 16 μM SP1. Few cells showed positive PI staining after SP1 treatment for 30 min (Fig. S3 in the supplemental material); however, the capsule was severely damaged at this time point (Fig. 6B). Thus, the cytoplasmic leaks caused by cell death may not be the cause of the SP1-induced capsule damage.

We used the *CAP59* deletion mutant (*cap59Δ*), which cannot form a capsule, to test whether SP1 damages only the capsule or damages other structures in addition to the capsule. As observed in the SEM images, there were no obvious morphological changes before or after SP1 treatment (Fig. 6C). Surprisingly, the *cap59Δ* mutant showed a higher sensitivity to SP1 compared with the wild-type strain (Table 1). Therefore, it is unlikely that SP1 leads to cell death directly via damage to the capsule.

### SP1 can be transported into the cytoplasm of C. neoformans cells.

**FIG 7:**
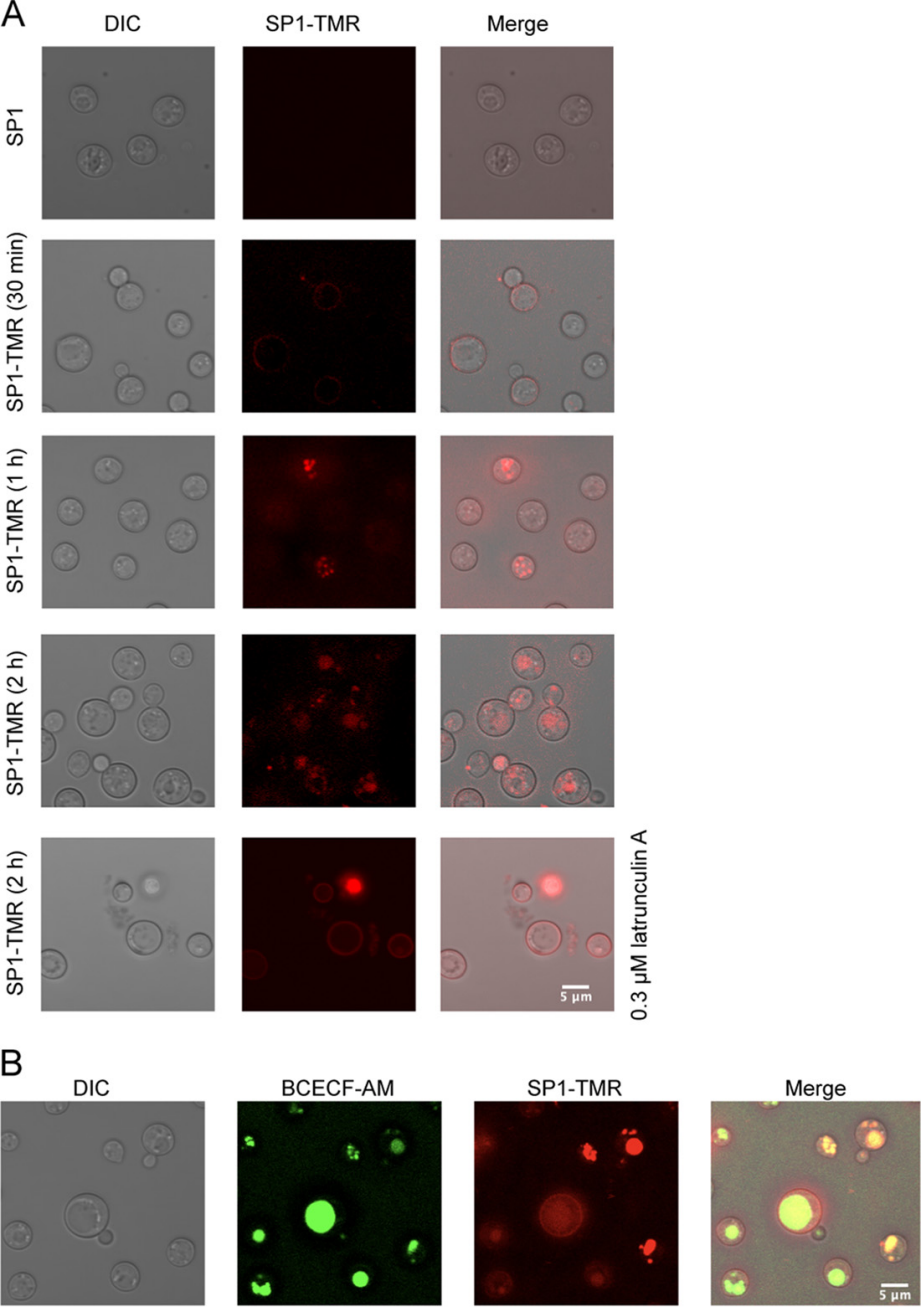
SP1 is transported to the cytoplasm of C. neoformans cells. (A) Confocal fluorescence microscopy of C. neoformans cells treated with TMR-labeled SP1. Representative images of C. neoformans (H99) cells that had been treated with 8 μM SP1, 8 μM SP1-TMR for 30 min, 1 h, 2 h and 2 h in the presence of 0.3 μM latrunculin A. (B) Fluorescence microscopy of C. neoformans cells which were incubated with 8 μM TMR-labeled SP1 for 2 h and BCECF-AM for 30 min.

To further address the SP1 mode of action, we examined the subcellular location of SP1 in C. neoformans using fluorescently labeled SP1. SP1 peptides were labeled with 5-carboxytetramethylrhodamine (TMR) and the treated cells were visualized by confocal microscopy. TMR-labeled SP1 was observed along the periphery of cells after a 30-min incubation (Fig. 7A), suggesting that the first target site of SP1 is on the surface of fungal cells. This observation is consistent with the effect of SP1 on capsules.

To examine whether TMR-labeled SP1 can be further translocated to the cytoplasm, we extended the incubation time to 1 h and 2 h. After 1 h of incubation, the SP1-TMR signal was spread out along the edge of the cells and fluorescent signals were also observed in the cytoplasm of some cells, implying that SP1 was able to enter the cells. After a 2-h incubation, the fluorescent signal of SP1-TMR was mostly detected inside the cell (Fig. 7A). Based on the signals of SP1-TMR displayed in Fig. 7A, we speculated that SP1 might be enriched in the vacuole after it was transferred into the cells. To further test this hypothesis, we adapted a fluorescent dye, BCECF-AM, to label the vacuole, which accumulates specifically in the vacuolar lumen (39). The co-staining of TMR-labeled SP1 and BCECF-AM showed that SP1 was located in the vacuole (Fig. 7B). It has been reported that the accumulation of AMPs in vacuoles is carried out by endocytosis (40). To verify whether SP1 translocates from the cell membrane to the vacuole via endocytosis, we treated cells with the actin inhibitor latrunculin A that can inhibit endocytosis. In the presence of latrunculin A, the fluorescent signal was mainly detected around the cell membranes, and little fluorescent signal was observed inside the cells, even after 2 h of incubation (Fig. 7A). Altogether, these data suggest that SP1 might first interact with ergosterol on the cell membrane and then translocate to the vacuole via endocytosis.

Next, we sought to determine whether the translocation of SP1 from the cell membrane to the vacuole is specific to C. neoformans. To this end, we examined the localization of SP1 in C. albicans. After treating C. albicans cells with TMR-labeled SP1 for 2 h, we observed that SP1 also translocated to the vacuole in C. albicans cells (Fig. S4 in the supplemental material). Thus, the translocation of SP1 is not specific to C. neoformans.

### SP1 induces apoptosis-like cell death of C. neoformans.

**FIG 8:**
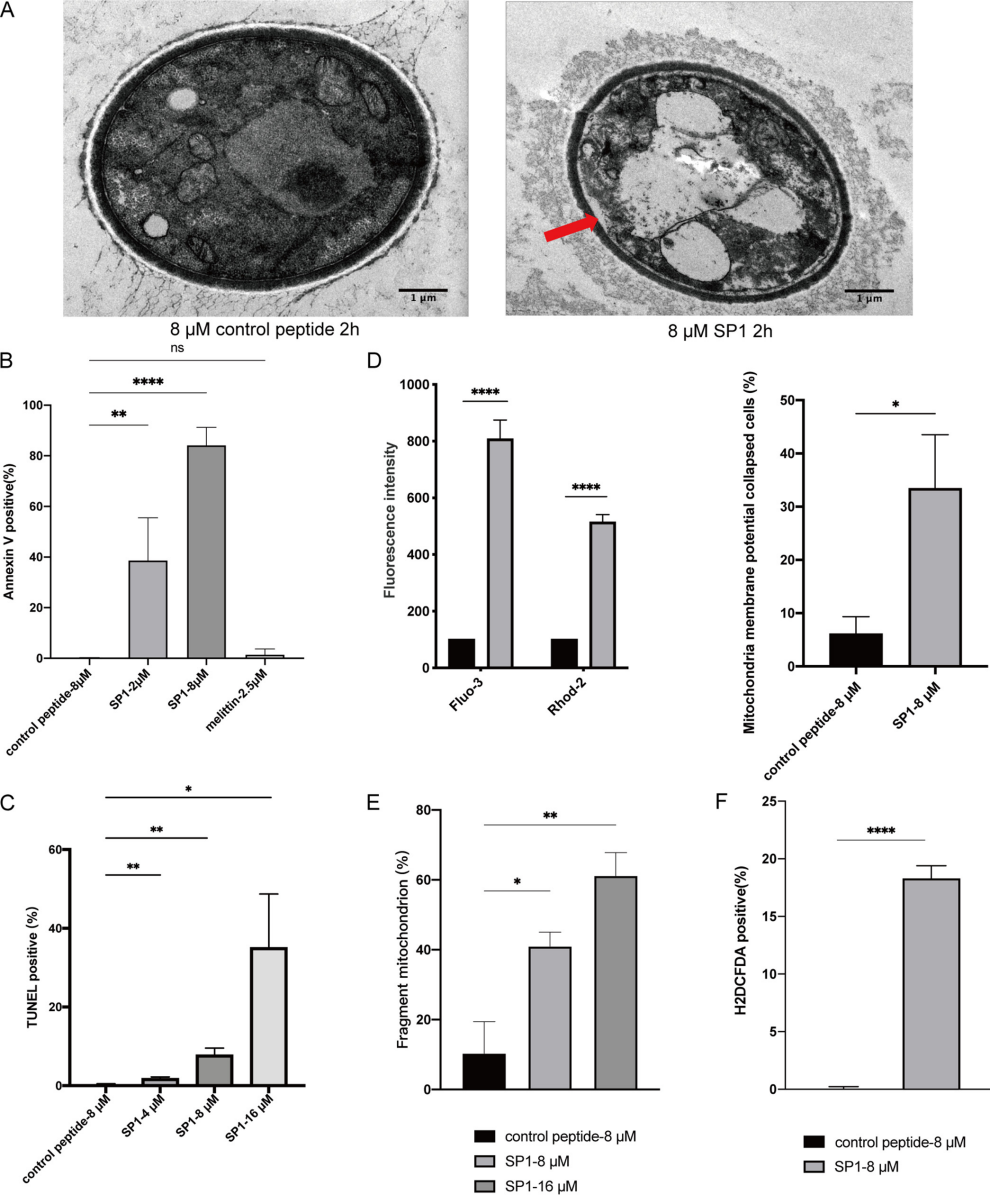
SP1 induces an apoptosis-like cell death of C. neoformans. (A) Transmission electron microscopic images of the cells after treating with 8 μM SP1 or control peptide for 2 h. The red arrow indicates the cell membrane invaginating. (B) Quantification of annexin V positive C. neoformans protoplast after SP1 treatment. The H99 protoplast was treated with control peptide, SP1, and melittin for 1 h. Flow cytometric analysis of annexin V staining was performed as described in Methods and Materials. Each experiment used around 30,000 protoplasts per treatment were detected by flow cytometry in three independent experiments, error bars represent the standard deviation of three experiments. (**P < 0.01, ****P < 0.0001). (C) Quantification of TUNEL-positive cells after SP1 treatment. The H99 cells were treated with control peptide or SP1 for 2 h and stained by TUNEL-PI. The staining cells were analyzed by flow cytometry. Error bars represent the standard deviation of three experiments (*P < 0.05, **P < 0.01). (D) Analysis of calcium ion levels and mitochondrial depolarization in the cells treated with SP1. The spectrofluorophotometric method was used to analyze cytosolic and mitochondrial calcium ion levels with Fluo-3-AM and Rhod-2-AM, respectively. H99 cells were treated with 8 μM SP1 or control peptide for 2 h, then the membrane potential of mitochondria was measured by JC-10 staining. Error bars represent the standard deviation of three experiments (*P < 0.05, ****P < 0.0001). (E) Distribution of fragmented mitochondria in the H99 cells with or without SP1treatment. The mitochondria were stained by Mitotracker Red CMXRos, and the distribution of fragmented mitochondria across populations were assessed after treating cells with 8 μM control peptide or 8 μM SP1. The percentage of cells containing fragment mitochondria was calculated from three independent experiments. Error bars represent the standard deviation of three experiments (*P < 0.05, **P < 0.01, ****P < 0.0001). (F) Accumulation of ROS in the H99 cells treated by SP1. The ROS levels were monitored by H2DCFDA staining in the cells treated with 8 μM control peptide or 8 μM SP1. Error bars represent the standard deviation of three independent experiments (****P < 0.0001).

To address how SP1 induces cell death of C. neoformans, we examined the morphology of C. neoformans after SP1 treatment for 2 h by transmission electron microscopy (TEM). The cell wall remained intact after SP1 treatment, suggesting that SP1 may not directly cause damage to the cell wall (Fig. 8A). However, as shown in Fig. 8A, SP1 treatment led to cell membrane invagination (red arrow). Moreover, compared with cells without SP1 treatment, some organelles, such as mitochondria, in SP1-treated cells appeared to be destroyed, and a large vacuole had formed (Fig. 8A).

To verify whether SP1 induces apoptosis-like cell death in the C. neoformans cells, we further examined several characteristic features of apoptosis after treating cells with SP1. Annexin V staining is a common method for detecting apoptotic cells. Annexin V is a protein that can bind to phosphatidylserine, a marker of apoptosis, when it is present on the outer leaflet of the plasma membrane. Because the presence of cell walls greatly affects annexin V staining, we used protoplasts for the annexin V binding assay. Only 2 μM SP1 was required to induce phosphatidylserine exposure on the inner side of the cell membrane in 40% of cells (Fig. 8B). Moreover, almost no phosphatidylserine exposure was detected in cells treated with 2.5 μM melittin (Fig. 8B). Combined with the previous observation that SP1 causes phosphatidylserine exposure (Fig. 8B and Fig. S5 in the supplemental material), these data suggest that SP1 could induce apoptosis-like cell death.

DNA fragmentation is another key feature of apoptosis. In the TUNEL assay, around 10% of cells displayed positive fluorescence following treatment with 8 μM SP1 for 2 h. In cells treated with 16 μM SP1 for 2 h, positive fluorescence was detected in nearly 35% of cells (Fig. 8C).

Subtle changes in Ca^2+^ concentrations can regulate and trigger apoptosis (41). Fluo-3 AM, a calcium indicator, was used to examine the concentration of calcium ions in the cytoplasm. SP1 treatment increased the concentration of calcium ions by 400% in the cytoplasm compared with the control peptide (Fig. 8D). Using the Rhod-2 AM staining assay that measures the mitochondrial calcium concentration, we found that SP1 treatment also increased the concentration of Ca^2+^ around 3-fold in mitochondria (Fig. 8D).

Mitochondrial calcium overload is a pro-apoptotic mechanism that promotes perturbations on the outer membrane of mitochondria. Mitochondrial membrane potential was monitored with the JC-10 mitochondrial membrane potential assay. Only ∼5% of cells lost their mitochondrial membrane potential following treatment with control peptides for 2 h. After treatment with SP1 for 2 h, the mitochondrial membrane potential collapsed in 35% of cells (Fig. 8D and Fig. S6 in the supplemental material). These data suggest that SP1 treatment results in mitochondrial membrane dysfunction.

It has been reported that C. neoformans cells cultured in DMEM form a tubular mitochondrial network to increase cellular ability against ROS stress (42). This phenomenon was observed in cells treated with the control peptide (Fig. 8E and Fig. S7 in the supplemental material). However, in cells treated with 8 μM and 16 μM SP1, 40% and 60% of cells entered a fragmented mitochondrial state, respectively, indicating mitochondrial destruction and inability to clear ROS (Fig. 8E). Moreover, we measured the concentration of ROS in the cytoplasm using the H2DCFDA assay. Compared with the control peptide-treated cells, treatment with 8 μM SP1 for 1 h led to a high level of ROS in ∼18% of cells (Fig. 8F).

Taken together, it is likely that SP1 can induce apoptosis-like cell death of C. neoformans. The induced apoptosis-like cell death was highly correlated with a change in Ca^2+^ concentrations, mitochondrial dysfunction, and elevated ROS levels caused by SP1 treatment.

### SP1 is nontoxic and nonhemolytic to mammalian cells.

To determine if SP1 exhibits toxicity to mammalian cells, the hemolytic activity of SP1 was tested in mammalian erythrocytes collected from healthy mice. The percentage of mammalian erythrocytes that underwent hemolysis was <16%, even in those treated with 32 μM SP1 (Fig. S8A in the supplemental material). Compared with AmB, SP1 showed little toxicity in HeLa cells at a concentration of 16 μM (Fig. S8B in the supplemental material). These results imply that SP1 is non-toxic and non-hemolytic to mammalian cells at its working concentration in C. neoformans.

## DISCUSSION

Effective antifungal agents are currently limited. Moreover, the emergence of large numbers of multi-drug-resistant fungi has necessitated the development of novel antifungal agents. Previous studies have confirmed that antifungal peptides are a class of novel antibiotics with efficient, broad-spectrum antifungal activity, good tissue penetration, and immunomodulatory activity (43). Our data show that micromolar levels of SP1, a peptide derived from budding yeast GAPDH protein, display promising antifungal activity against C. neoformans and C. gattii, including C. neoformans strains that are resistant to fluconazole and amphotericin B. Additionally, SP1 is nontoxic and nonhemolytic to mammalian cells. Therefore, SP1 is a potential candidate for antifungal drug development.

To our knowledge, SP1 is the only peptide derived from GAPDH that can efficiently and specifically kill Cryptococcus. Moreover, SP1 homologs from the GAPDH protein of other yeast species do not have this ability, even though the SP1 homologous peptide from C. albicans shares 78% sequence identity with SP1 from S. cerevisiae. This indicates that the specific amino acid sequence of SP1 is essential to kill Cryptococcus.

Many peptides kill cells by forming pores within the cell membrane. Based on our observations, SP1 does not form pores on the membrane. In our lipid vesicle experiments, SP1 induced leakage of POPC/POPE/Erg lipid vesicles, but did not induce leakage of POPC:POPG or POPC:Chl vesicles. The *erg4Δ* mutant showed resistance to SP1-mediated killing. We also observed that SP1 treatment caused decreased filipin III staining of ergosterol within cell membranes. Thus, SP1 appears to interact with ergosterol in the cell membrane, however the presence of other SP1 targets within the membrane cannot be excluded. We observed that SP1 was translocated from the membrane to the vacuole by endocytosis and may have accumulated in the vacuole following the vacuole fusion process. It has been reported that vacuolar fusion in fungi is dependent on ergosterol (44). Furthermore, in fungi, the vacuoles store calcium and this calcium accounts for more than 90% of the total cell volume (45). Because calcium ions in vacuoles mainly exist within complexes (46), SP1 may promote the release of free calcium ions from the vacuoles into the cytoplasm, leading to apoptosis-like cell death. After SP1 treatment, we observed an increase in the cytoplasmic calcium ion concentration (Fig. 8D). Moreover, we detected a substantial increase in the mitochondrial calcium concentration, which led to a loss of mitochondrial membrane potential. This could have been caused by reverse transport of calcium ions into mitochondria because of high cytoplasmic calcium concentrations.

We observed that SP1 treatment damages the C. neoformans capsule. This significant change in the appearance of the capsule could reflect either a structural change or protein deposition in the capsule. However, SP1 was able to kill the C. neoformans *cap59Δ* mutant which lacks capsule more efficiently than the wild type cells. Thus, it is unlikely that SP1 killed C. neoformans by a direct effect on the capsule. It is well known that the capsule contributes significantly to the pathogenesis of cryptococcal disease (47). The capsule of C. neoformans has been reported to inhibit phagocytosis during infection (48). The effect of SP1 on the capsule could be an advantage for the treatment of cryptococcosis.

It remains unclear why SP1 was effective at killing only Cryptococcus, but not the other fungal pathogens, such as C. albicans and A. fumigatus. We observed that SP1 can be translocated to the vacuole in C. albicans cells after treatment (Fig. S4 in the supplemental material). Thus, the translocation itself may not be responsible for the killing of Cryptococcus species. It is plausible that SP1 may interact with Cryptococcus-specific proteins during translocation to induce the release of intracellular calcium ions. The interaction between Cdc50 (the β-subunit of membrane lipid translocase) and Crm1 (a putative mechanosensitive calcium channel protein in the endoplasmic reticulum) maintains cellular calcium homeostasis in C. neoformans (49, 50). Therefore, it would be interesting to investigate whether SP1 affects the corresponding calcineurin pathway. In addition, although the SP1-induced capsule damage was not the cause of the observed cell death of C. neoformans, the damage to the capsule could have induced a specific compensatory capsule synthesis pathway. Proteins involved in the compensatory pathway could be targets of SP1, and this mechanism could lead to C. neoformans cell death. This hypothesis would also explain the higher sensitivity of the *cap59Δ* mutant to SP1. The relationship between SP1 and the transcriptional factors regulating capsule synthesis, such as Skn7 and Fzc1 (51), will be investigated in future studies.

In summary, our results show that SP1 has antifungal activity against C. neoformans *in vitro*; thus, this peptide may be clinically useful for C. neoformans infections. However, further work is needed to assess the effects of SP1 *in vivo.* Future studies should focus on potential design modifications of SP1 that might increase its stability and efficiency against C. neoformans *in vivo*.

## MATERIALS AND METHODS

### Yeast and bacteria strains.

C. neoformans strains JEC21, H99, W82, W83, *cap59*Δ and C. gattii strains W1010, W1011, W1012, W2520 were gifts from Linqi Wang at Institute of Microbiology, Chinese Academy of Sciences. The *erg4*Δ, *fzc51*Δ, *atf1*Δ, *fzc9*Δ, *gat5*Δ, *liv4*Δ, and *sip4*Δ mutants in the H99 background were obtained from the Fungal Genetics Stock Center (FGSC, http://www.fgsc.net/crypto/crypto.htm). C. neoformans strains R272 and WM179 were kindly shared by Min Chen at Shanghai Changzhen Hospital. C. albicans (SC5314) and Aspergillus fumigatus (YJ407) were gifts from Guanghua Huang and Cheng Jin at the Institute of Microbiology, Chinese Academy of Sciences, respectively. The S. cerevisiae (BY4741) and bacteria Pseudomonas aeruginosa, Escherichia coli, Micrococcus luteus, and Staphylococcus aureus were stocks in our lab. *fzc51*Δ and *atf1*Δ strains showed AmB resistance (51). *fzc9*Δ, *gat5*Δ, *liv4*Δ, and *sip4*Δ strains showed FLC resistance (51).

### Peptide synthesis.

The peptides SP1, control peptide, and SP1-TMR were synthesized from Genscript (Nanjing, China) with a purity of 98%. The SP1 homologous peptides from C. neoformans, C. albicans, and A. fumigatus were synthesized from GL biochem (Shanghai, China) with a purity of 95%. The purity was further confirmed by HPLC-mass spectrometry. The amino acid sequence of SP1 is IRIAINGFGRIGRLVLRLALQRKDIEVVA, and the amino acid sequence of control peptide is IRIAINGFGRIGRPPPRPPPQRKDIEVVA. The amino acid sequences of SP1 homologous peptides from C. neoformans, C. albicans, and A. fumigatus are VKVGINGFGRIGRIVLRNAIEHGDLEVVA, IKIGINGFGRIGRLVLRVALGRKDIEVVA, and PKVGINGFGRIGRIIGLNSLSHGLVDVVA, respectively.

### Drugs and AMPs.

AmB, FLC, Flucytosine, and Caspofungin were purchased from Meilunbio (Dalian, China). Melittin was purchased from Aladdin (Shanghai, China).

### Antifungal activity assay.

The *in vitro* MIC of SP1 was assessed as described in the CLSI M27-A4 (52). To determine the MIC of SP1 for C. neoformans, we adopted RPMI 1640 medium with 0.165 M MOPS buffer and cultured yeast cells at 37°C for 72 h. The value of the MIC was defined as the lowest concentration of drugs showing no yeast growth. The MIC was determined by three independent repeats, each repeat in quintuplicate.

### Time-killing experiment.

The time-killing experiment was performed as described (17). C. neoformans H99 cells were cultured in RPMI 1640 medium overnight, and the concentration was adjusted to 3 × 10^7^ colony-forming units (CFU)/mL. After adding different drugs, control peptide or SP1, add an equal volume of sterile ultrapure water as a growth control, cells were cultured at 37°C. Aliquots were taken out to wash and serially diluted 1:10 in RPMI 1640 medium to final concentration of nearly 300 CFU/mL, and plate on the YPD medium at the corresponding time points. After incubating at 37°C for 48 h, colonies were counted to calculate the survival rate. The percentage of CFU of growth control was set as 100%. The percentage of recovering CFU on plates was plotted against time. The data were obtained from three independent experiments.

### Secondary structure analysis and structural modeling.

The theoretical isoelectric point and molecular mass of SP1 were estimated with expasy (http://web.expasy.org/peptide_mass/). The secondary structure was predicted by the GOR method (https://npsa-prabi.ibcp.fr/cgi-bin/npsa_automat.pl?page=npsa_gor4.html) (53). The model of the structure of SP1 was built by homology modeling using the SWISS-MODEL server (54). The structure of GAPDH (accession no. 2i5p.1.A) was used as a template for modeling (55–57).

### Calcein leakage assay.

These experiments were performed as previously described (34). Briefly, phospholipid vesicles were prepared from POPC: POPE (at a ratio of 1:4), POPC/POPE/Erg (at a ratio of 5:4:3), and POPC with 40% cholesterol, which mimics the cell membranes of bacterial, fungal, and mammalian cells, respectively. The resulting lipid film was suspended in Tris buffer (20 mM Tris, 100 mM NaCl, and 40 mM calcein, pH 7.4). Extravehicular buffer containing lipid vesicles with entrapped calcein was read in a fluorimeter using excitation at 490 nm and emission at 520 nm. After stabilization of calcein fluorescence, SP1 was added and the fluorescence enhancement was measured 10 min after each addition. The 10% Triton X-100 was used to disrupt lipid vesicles for obtaining the maximum fluorescence intensity. The percent leakage was calculated using the following equation: percentage leakage = (F − F_0_)/(F_T_ − F_0_) × 100, where F is the fluorescence intensity after SP1 addition, F_0_ is the basal fluorescence intensity, and F_T_ is the maximum fluorescence intensity after the addition of Triton X-100.

### Flow cytometry analysis.

H99 cells were cultured in RPMI 1640 medium (Sigma, Shanghai) with 0.165 M MOPS (Sigma, Shanghai) overnight and diluted in fresh medium to an OD_600_ of 1. For the Annexin V-PI assay, the yeast protoplast preparation kit (BestBio, Shanghai) was used to prepare the protoplasts. After incubation with control peptide, SP1 or 2.5 μM melittin, the protoplasts were stained by Annexin V-FITC/PI apoptosis kit (MultiScience Biotech, Hangzhou) according to the manufacturer’s instructions. The TUNEL assay was performed by using the Apoptosis Detection Kit (Yeasen, Shanghai) according to the manufacturer’s instructions. For JC-10 staining, H99 cells were stained by 30 μM JC-10 (Yeasen, Shanghai) and 0.02% Pluronic F-127(Beyotime, Shanghai) at 37°C for 30 min. The intracellular ROS was stained by the incubation with 5 μM H2DCFDA(MCE, Shanghai) at 37°C for 30 min. The stained samples were measured by BD FACSCalibur as described (58). The data were analyzed by FlowjoX. All experiments were repeated three times independently.

### Measurement of the concentrations of intracellular and mitochondrial calcium ion.

The Fluo-3 AM and Rhod-2 AM (Yeasen, Shanghai) were used to measure the cytoplasmic and mitochondrial Ca^2+^ levels as described (58). The SP1 or control peptide treated cells were washed three times with Krebs buffer (132 mM sodium chloride, 4 mM potassium chloride, 1.4 mM magnesium chloride, 6 mM glucose, 10 mM HEPES, 10 mM sodium bicarbonate, pH 7.2), followed by the incubation with 5 μM Fluo-3 AM or 5 μM Rhod-2 AM at 28°C for 30 min. The fluorescence intensities of Fluo-3 AM and Rhod-2 AM were measured with a spectrofluorophotometer (Synergy).

### CD spectroscopy.

CD experiments were performed by using CD spectroscopy (Applied photophysics, United Kingdom) to determine the secondary structure of SP1 at 15°C, 25°C, 35°C, 45°C, 55°C in a 1-mm quartz cell. The peptide was dissolved in 50% trifluoroethyl alcohol (TFEA) (Macklin, Shanghai). NaOH or hydrochloric acid were used to adjust pH. CD spectra were acquired using a measurement ranging from 180 to 260 nm, and a 0.5 nm/min scanning speed was used. All measurements were repeated three times. The analysis of the secondary structure content was performed using the CDSSTR method at the DICHROWEB website (59–61), and the reference data set SP175 was used to analyze the spectrum data (62).

### Confocal laser-scanning microscopy of stained cells.

To image the localization of SP1-TMR, yeast cells were cultured in RPMI 1640 medium with 0.165 M MOPS. Cells were incubated with SP1 for 2 h, and SP1-TMR, respectively, for 30 min, 1 h, or 2 h. For the BCECF-AM co-staining, we used 5 μM BCECF-AM (Beyotime, Shanghai) to stain the cells which were treated by SP1-TMR for 2 h. To test the effects of endocytosis, we added 0.3 μM latrunculin A and 8 μM SP1-TMR in the yeast cultures for a 2-h incubation. All fluorescent images were collected with the confocal microscope (Olympus IX81) at a magnification of ×100 (oil immersion). To observe the effect of SP1 on the capsule of C. neoformans, the H99 cells were treated with control peptide and SP1 for 60 min and then stained by the 50% India ink (SECOMA, Ohio). Images were taken using the same confocal microscope described above. To observe the morphology of mitochondria, H99 cells with or without SP1 treatment were incubated with Mitotracker Red CMXRos (Yeasen, Shanghai) at a final concentration of 0.5 μM at 37°C for 15 min, followed by three times wash and fixation using 4% formaldehyde-PBS. The fluorescent images of mitochondria were acquired on the confocal microscope (Leica SP8). At least 200 cells per sample were categorized based on their tubular or fragmented mitochondria morphologies. To quantify the fluorescent staining of ergosterol by filipin III, H99 cells with different treatment were stained by filipin III as described (37). The fluorescent images were acquired by the IX81 confocal microscope described above. The ImageJ was used to measure the mean fluorescent intensity. The statistical analysis of fluorescent intensity was based on three independent experiments. In each experiment, at least 100 cells were counted in three different visual fields.

### Scanning electron microscopy and transmission electron microscopy.

For the SEM, the treated H99 cells were washed with PBS and fixed by 2.5% glutaraldehyde. The specimens for SEM were prepared according to the standard sample preparation method as described (63). The gold-coated samples were scanned by the SEM (Hitachi SU8010). For the Transmission Electron Microscopy, H99 cells cultured in RPMI 1640 with 0.165 M MOPS were treated with SP1 or control peptide. After wash and fixation, the cells were embedded as described (64). The embed samples were cut to 60-nm slices by an ultra-thin slicer. Slices were harvested on a supported film and observed using JEM-1400 (120 kV) transmission electron microscope.

### Hemolytic activity against mammalian erythrocytes.

To assess the cytotoxicity of SP1 against mammals, hemolytic activity was evaluated by the percentage of hemolysis in a 4% suspension of mammalian red blood cells (mRBCs) in various concentrations (from 4 μM to 64 μM) of SP1. Briefly, the mRBCs were washed three times with PBS. Aliquots of mRBC suspensions were added to 96-well microtiter plates, and SP1 was then mixed into each well. After incubating the mixtures for 4 h at 37°C, the mixtures were centrifuged and the resulting supernatants were transferred to new 96-well microtiter plates. The absorbance of the liquid supernatants was measured at 540 nm by using a microtiter ELISA Reader. Ultra-pure water and PBS were used as positive control and negative control in the hemolysis test, respectively. The percentage of hemolysis was calculated by employing the following equation: percentage hemolysis = [(Abs_540_ in the compound solution − Abs_540_ in PBS)/(Abs_540_ in H_2_O − Abs_540_ in PBS)] × 100%.

### Cytotoxicity tests.

HeLa cell line is a gift from the Min Fang (Chinese Academy of Sciences, Beijing, China). HeLa cells were cultured in DMEM medium supplemented with 10% fetal bovine serum at 37°C in a humidified atmosphere with 5% CO_2_. HeLa cells were treated at 37°C with SP1 at different concentrations (4, 16, 32, or 64 μM) for 6 h, and the same densities of the control peptide were added as negative controls. The cell death of HeLa cells after treatment was monitored by PI staining assay using flow cytometry with the MOFLO XDP (Beckman Coulter, Germany). The toxicity of SP1 to HeLa cells was evaluated by the proportion of PI-positive cells.

### Method of statistical analysis.

All statistical analyses were performed based on the data collected from three independent experiments. The significance of the difference was tested by Student's *t* test, and the error bars on the graphs represented the standard deviation of a data set.
