# Supplementary material for: A Peptide from Budding Yeast GAPDH Serves as a Promising Antifungal against Cryptococcus neoformans
Source: Microbiol Spectr. 2022 Jan 12;10(1):e00826-21. doi: 10.1128/spectrum.00826-21 (PMC8754130; doi:10.1128/spectrum.00826-21)
Supplement: SUPPLEMENTAL FILE 1 — Supplemental material. Download Spectrum00826-21_Supp_1.pdf, PDF file, 3.4 MB [file spectrum00826-21_supp_1.pdf]

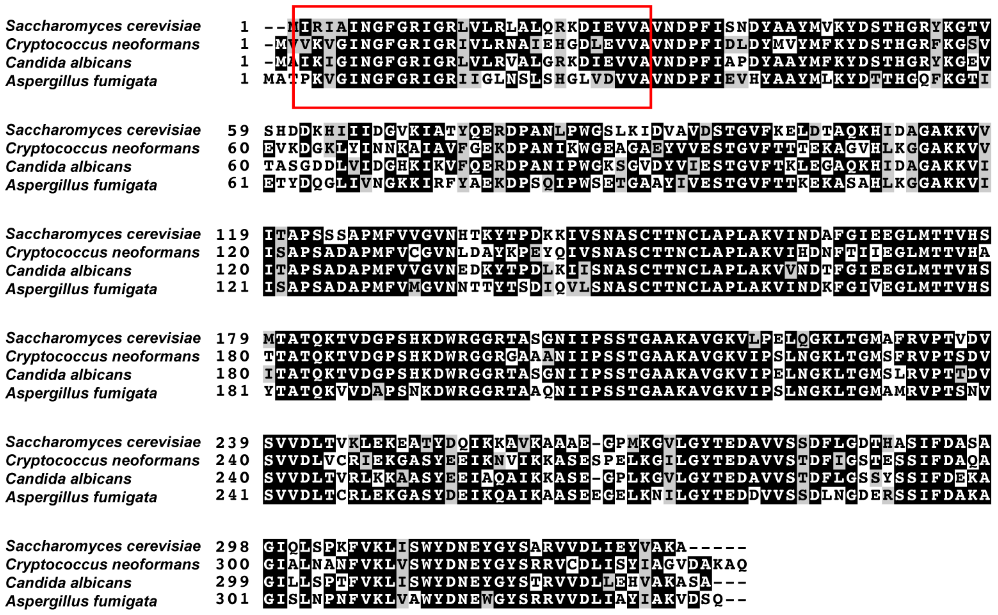

**Fig S1.** Sequence alignment GAPDH from *Saccharomyces cerevisiae* , *Cryptococcus neoformans, Candida albicans* and *Aspergillus fumigatus*, the red box showed the amino acid sequences of SP1 and SP1 homolgous peptides from different species.

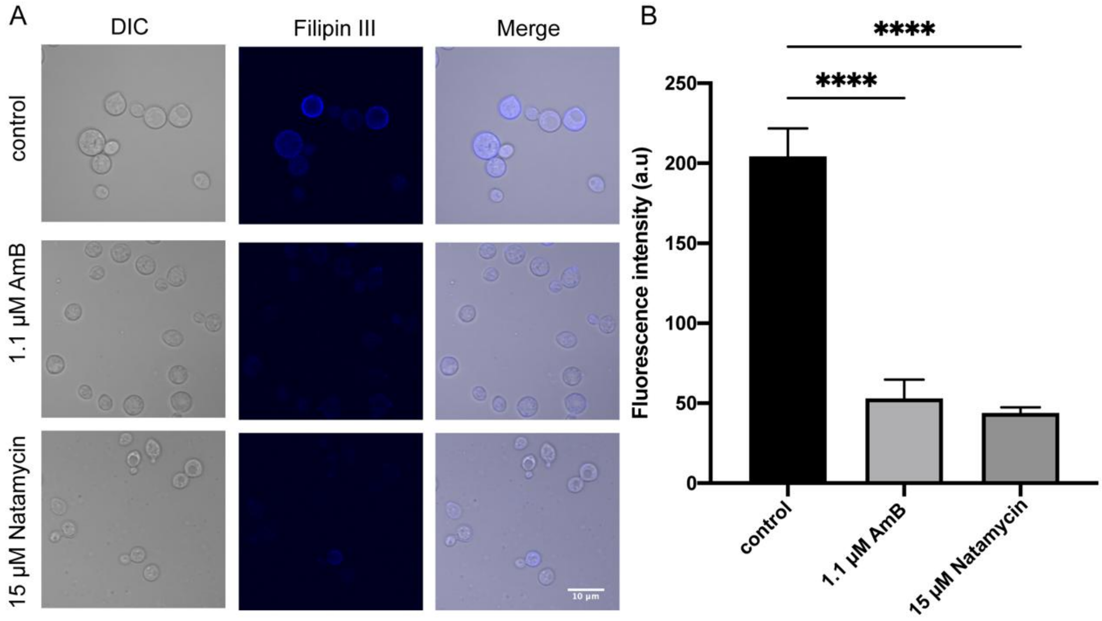


**Fig S2**. (A)Confocal fluorescence microscopy of filipin III stained *C.neoformans* cells. The *C. neoformans* (H99) cells was treated with 1.1 μM AmB or 15 μM Natamycin for 1 hour before acquiring the images.(B) Mean fluorescence intensity of filipin III stained *C.neoformans* (H99) cells treated with 1.1 μM AmB or 15 μM Natamycin for 1 hour. The fluorescence intensity was calculated by ImageJ as described in Methods and Materials, error bars represent the standard deviation of three experiments (*$p$<0.0001) .

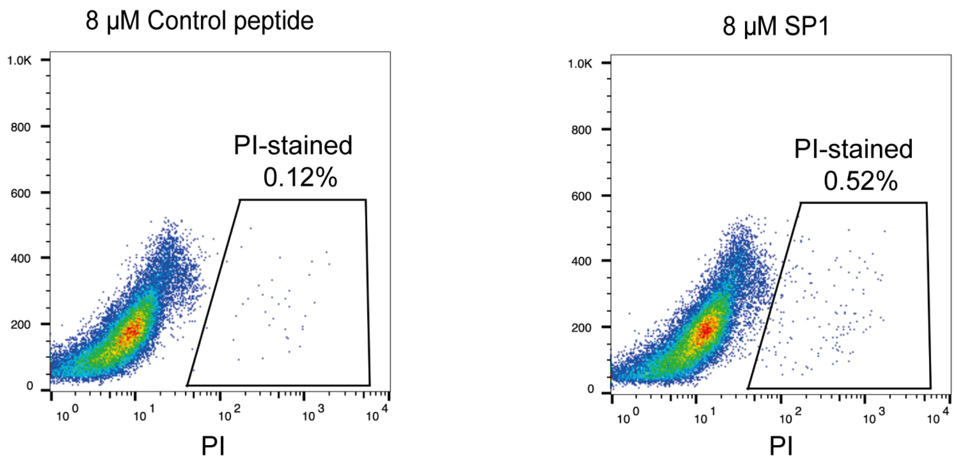


**Fig S3.** Flow cytometry analysis of PI-stained *C. neoformans.* H99 cells were treated with 8 µM SP1 or control peptide for 30 min, then stained by PI for flow cytometry. Around 30,000 cells per treatment were detected by flow cytometry in three independent experiments.

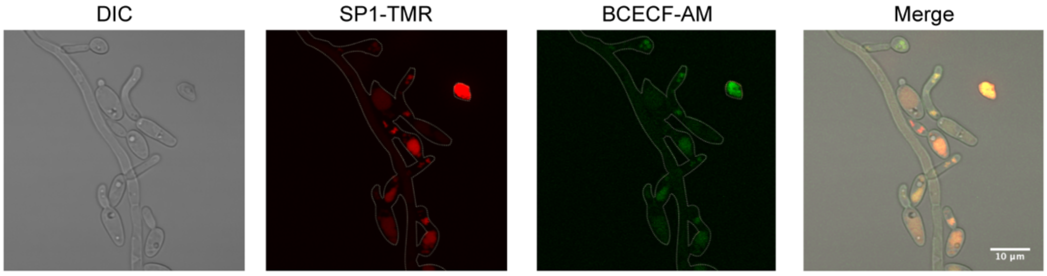


**Fig S4.** Fluorescence microscopy of *C. albicans* cell which were co-incubated with 8 μM TMR-labeled SP1 2 h and BCECF-AM for 30 min.

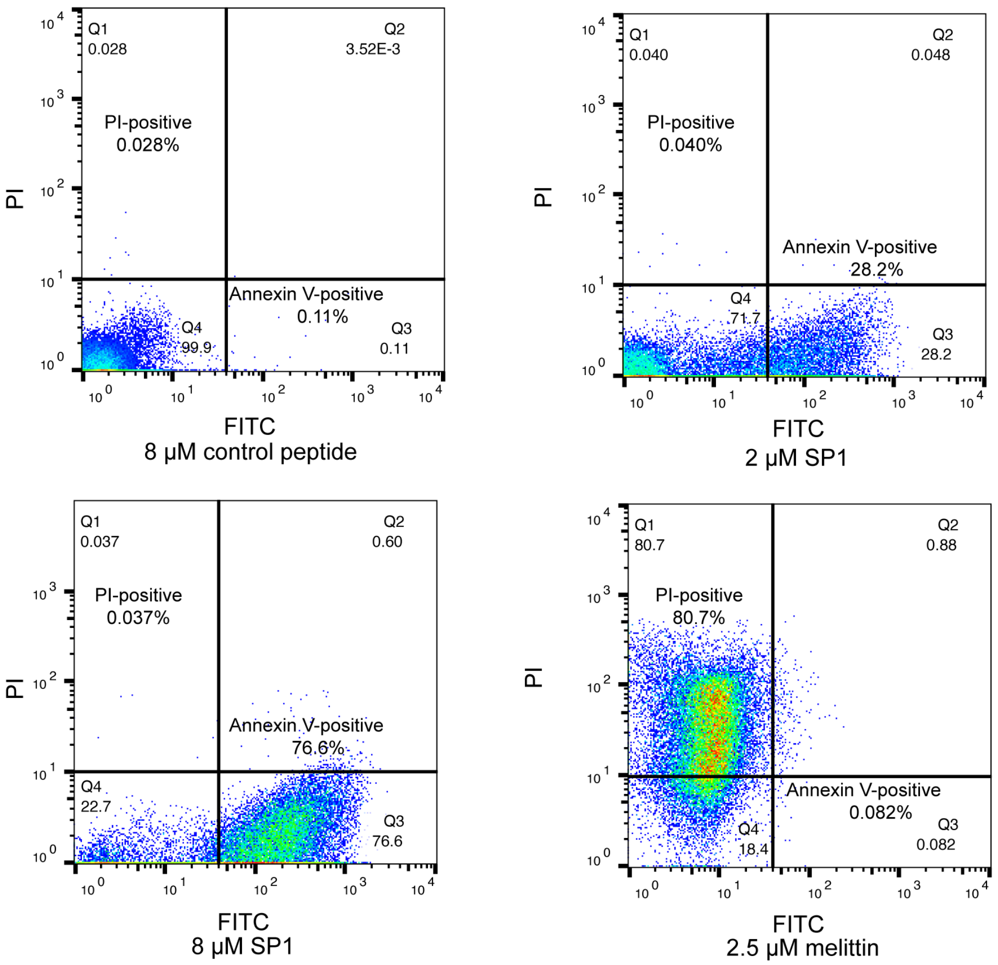


**Fig S5.** Flow cytometry analysis of Annexin V-PI stained protoplast of *C. neoformans*. The H99 protoplast was treated with control peptide, SP1, and melittin for 1 hour. Flow cytometric analysis of annexin V-PI double-stained was performed as described in Methods and Materials. The upper left quadrant (Q1) indicates PI positive cells, the lower right quadrant (Q3) denotes Annexin V positive cells. Around 30,000 protoplasts per treatment were detected by flow cytometry in three independent experiments.

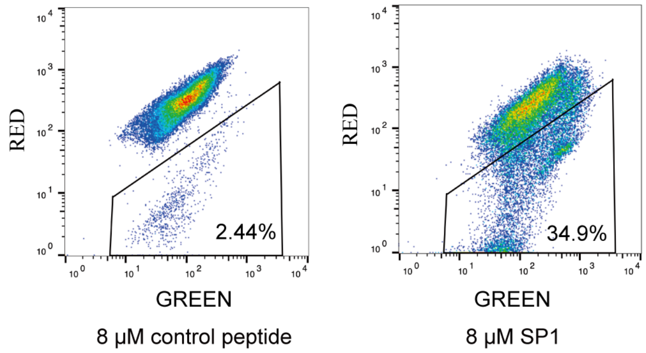


**Fig S6.** Mitochondrial depolarization was detected by JC-10 staining. H99 cells were treated with SP1 (8 μM) at 37 °C for 2 h，and 8 μM control peptide was used as the negative control.

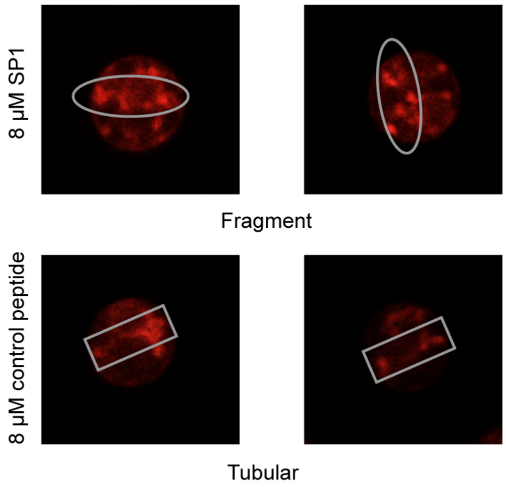


**Fig S7.** Morphological characteristics of Mitochondria after SP1 treated. H99 cells treated by 8 μM control peptide or 8 μM SP1. The mitochontria were stained by Mitotracker Red CMXRos. The gray oval and rectangle represents the typical fragmental or tubular mitochondria respectively.

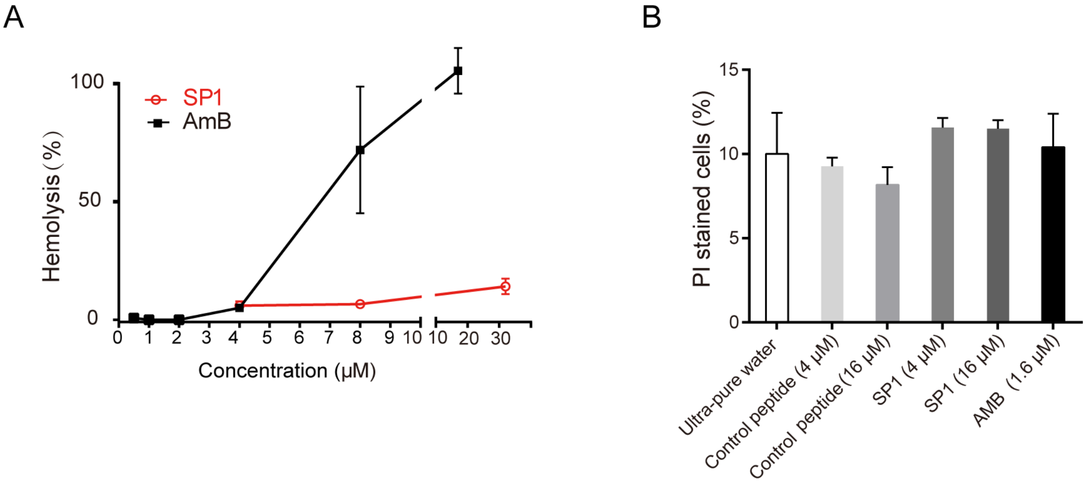


**Fig S8.** Hemolytic assay and cytotoxicity test of SP1. (A) The hemolytic effects of SP1 and AmB on mouse erythrocyte cells. (B) Bar plot shows the percentage of PI-stained of Hela cells that had been treated with water, AmB, or different concentrations of SP1 or control peptide, as assessed by flow cytometry. The error bars represent the SD form three independent experiments, performed in triplicate.

## <Chromatogram>

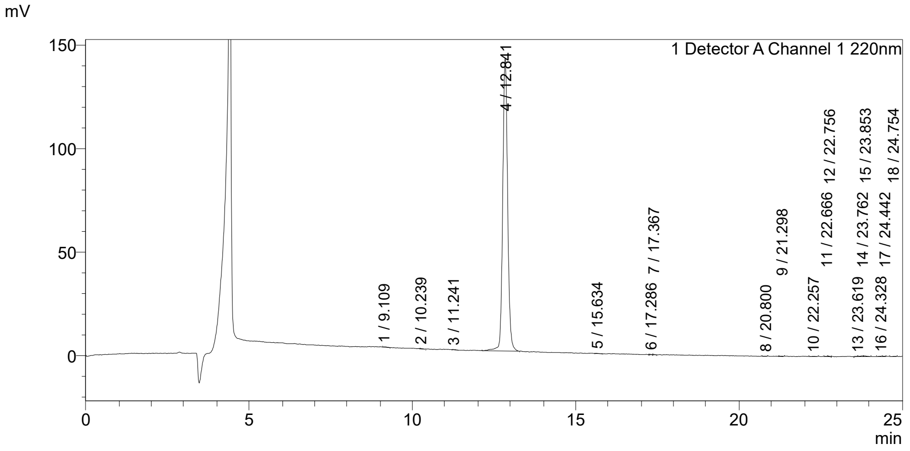


## <Peak Table>

Detector A Channel 1 220nm

| Peak# | Ret. Time | Area | Height | Area% |
|---|---|---|---|---|
| 1 | 9.109 | 1453 | 107 | 0.096 |
| 2 | 10.239 | 1001 | 181 | 0.066 |
| 3 | 11.241 | 1059 | 157 | 0.070 |
| 4 | 12.841 | 1482976 | 141552 | 98.467 |
| 5 | 15.634 | 1174 | 141 | 0.078 |
| 6 | 17.286 | 1220 | 243 | 0.081 |
| 7 | 17.367 | 1069 | 204 | 0.071 |
| 8 | 20.800 | 1090 | 176 | 0.072 |
| 9 | 21.298 | 1366 | 207 | 0.091 |
| 10 | 22.257 | 1258 | 163 | 0.084 |
| 11 | 22.666 | 1039 | 177 | 0.069 |
| 12 | 22.756 | 1153 | 254 | 0.077 |
| 13 | 23.619 | 1374 | 203 | 0.091 |
| 14 | 23.762 | 2978 | 409 | 0.198 |
| 15 | 23.853 | 2747 | 360 | 0.182 |
| 16 | 24.328 | 1011 | 199 | 0.067 |

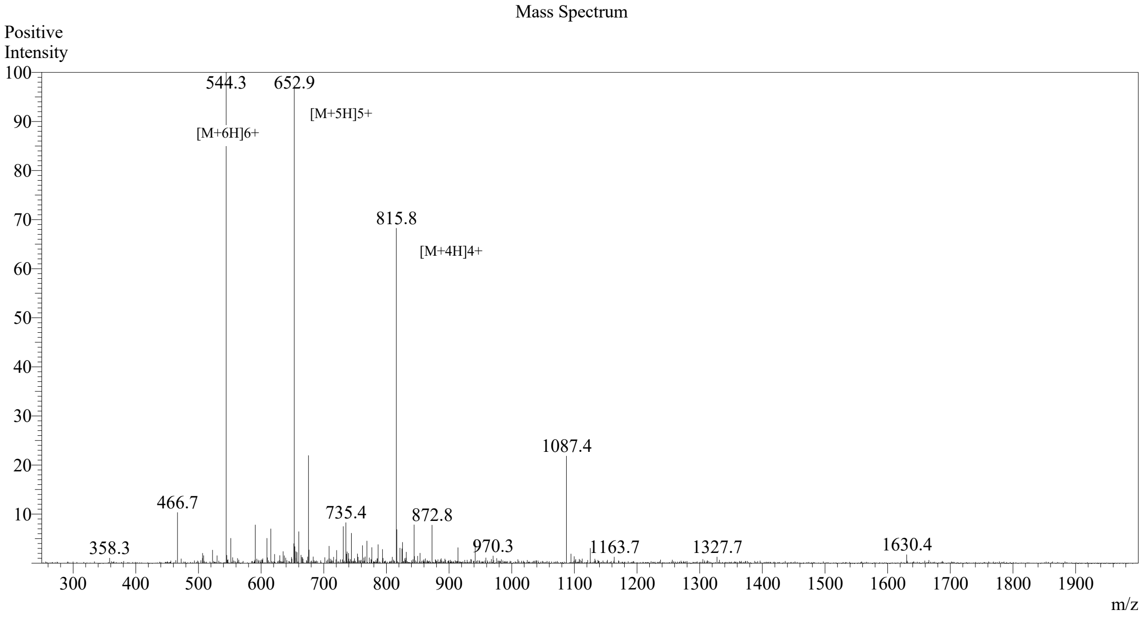


**Fig S9.** HPLC and mass-spectrometry of SP1.

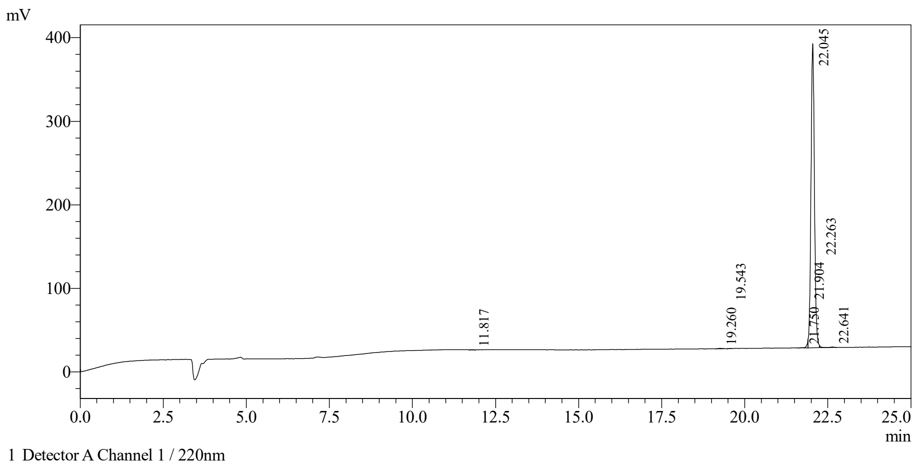


Peak Table

Detector A Channel 1 220nm

| Peak# | Ret. Time | Area | Height | Area % |
|---|---|---|---|---|
| 1 | 11.817 | 1418 | 228 | 0.053 |
| 2 | 19.260 | 5030 | 655 | 0.188 |
| 3 | 19.543 | 1253 | 277 | 0.047 |
| 4 | 21.750 | 2904 | 358 | 0.109 |
| 5 | 21.904 | 22792 | 8153 | 0.852 |
| 6 | 22.045 | 2632526 | 364095 | 98.372 |
| 7 | 22.263 | 5505 | 1980 | 0.206 |
| 8 | 22.641 | 4655 | 529 | 0.174 |
| Total | | 2676083 | 376275 | 100.000 |

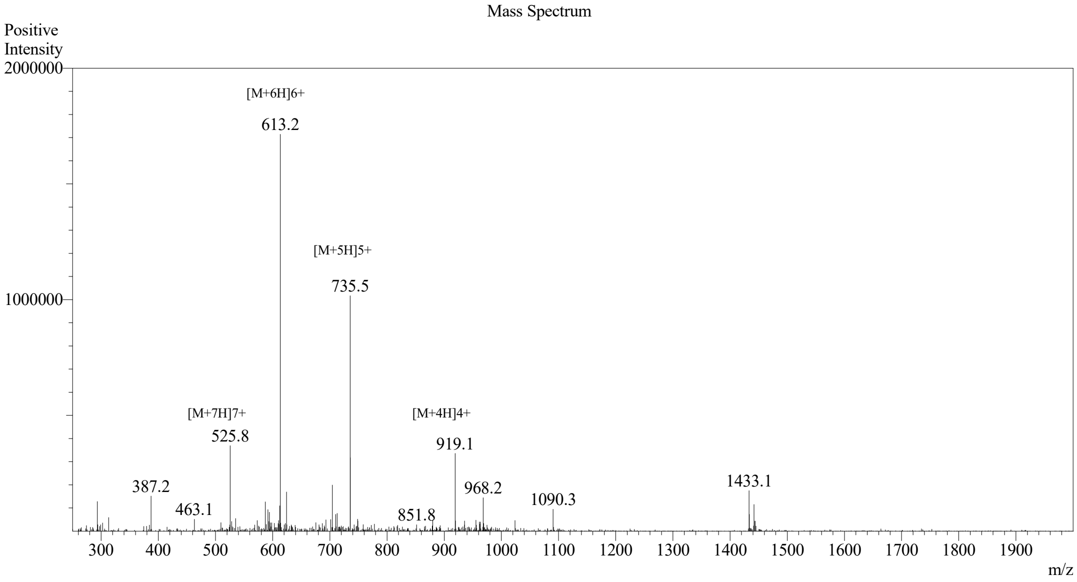


**Fig S10.** HPLC and mass-spectrometry of SP1-TMR.

**<Chromatogram>**

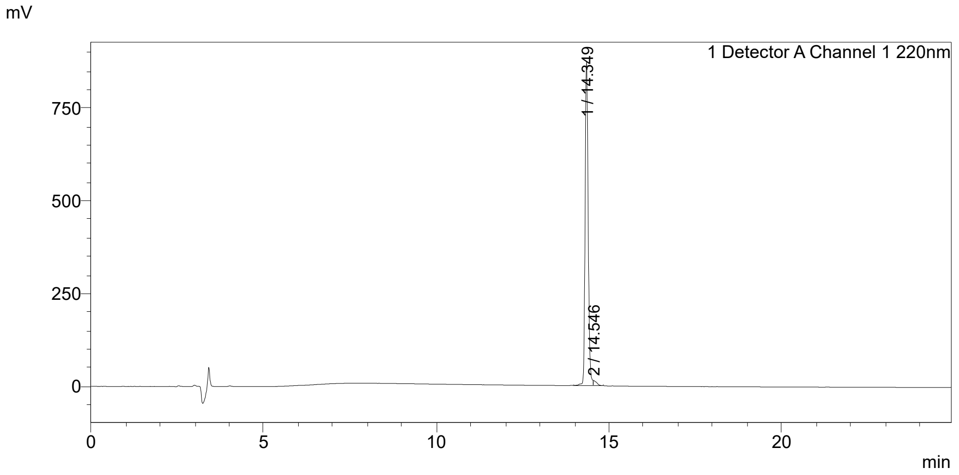


**<Peak Table>**

Detector A Channel 1 220nm

| Peak# | Ret. Time | Area | Height | Area% |
|---|---|---|---|---|
| 1 | 14.349 | 5388302 | 874862 | 98.311 |
| 2 | 14.546 | 92581 | 14394 | 1.689 |
| Total | | 5480883 | 889256 | 100.000 |

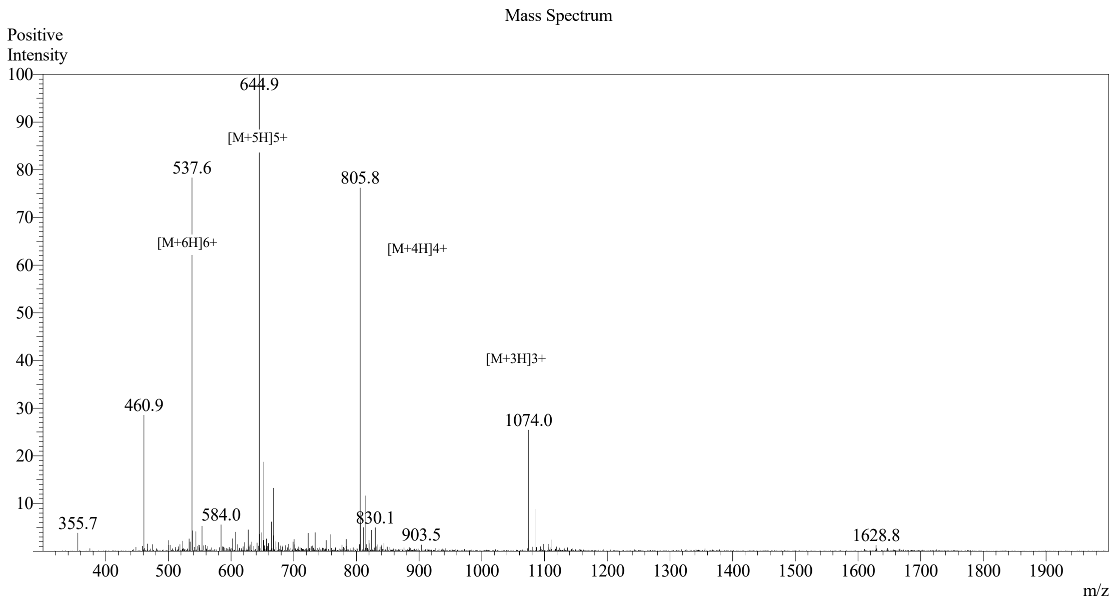


**Fig S11.** HPLC and mass-spectrometry of control peptide.

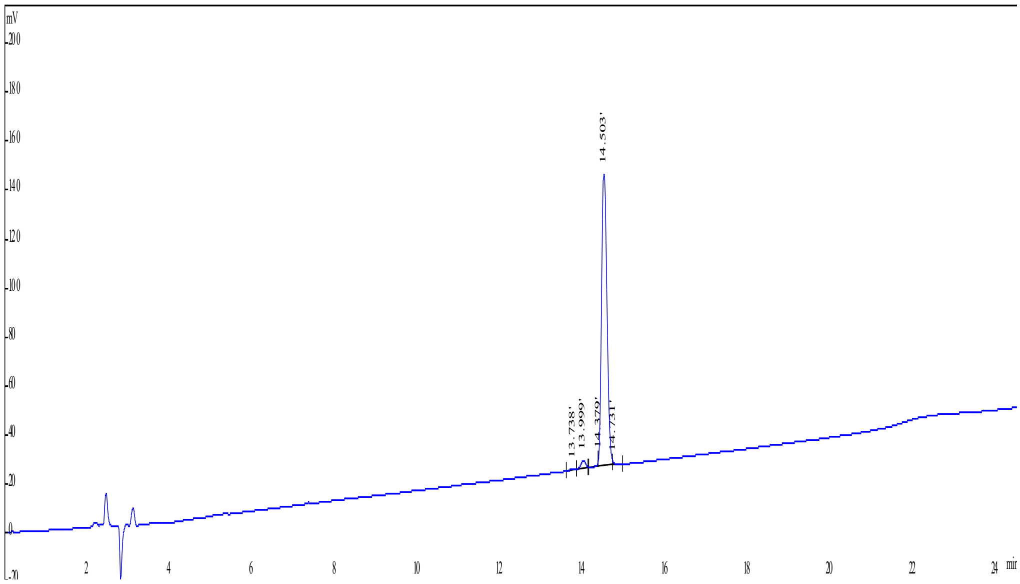


| Rank | Time | Conc. | Area | Height |
|---|---|---|---|---|
| 1 | 13.738 | 0.2963 | 2974 | 457 |
| 2 | 13.999 | 2.1314 | 21392 | 3001 |
| 3 | 14.379 | 0.3596 | 3609 | 3092 |
| 4 | 14.503 | 96.7229 | 970755 | 119206 |
| 5 | 14.731 | 0.4898 | 4916 | 765 |
| Total | | 100 | 1003646 | 126521 |

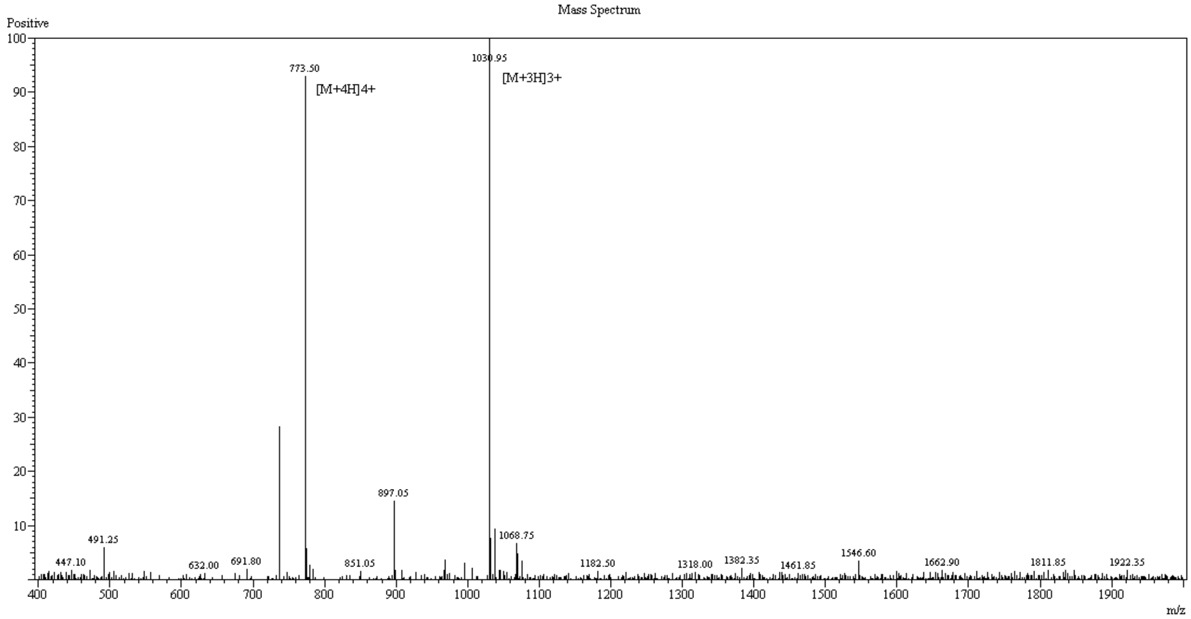


**Fig S12.** HPLC and mass-spectrometry of SP1 homologus peptide from *C.neoformans.*

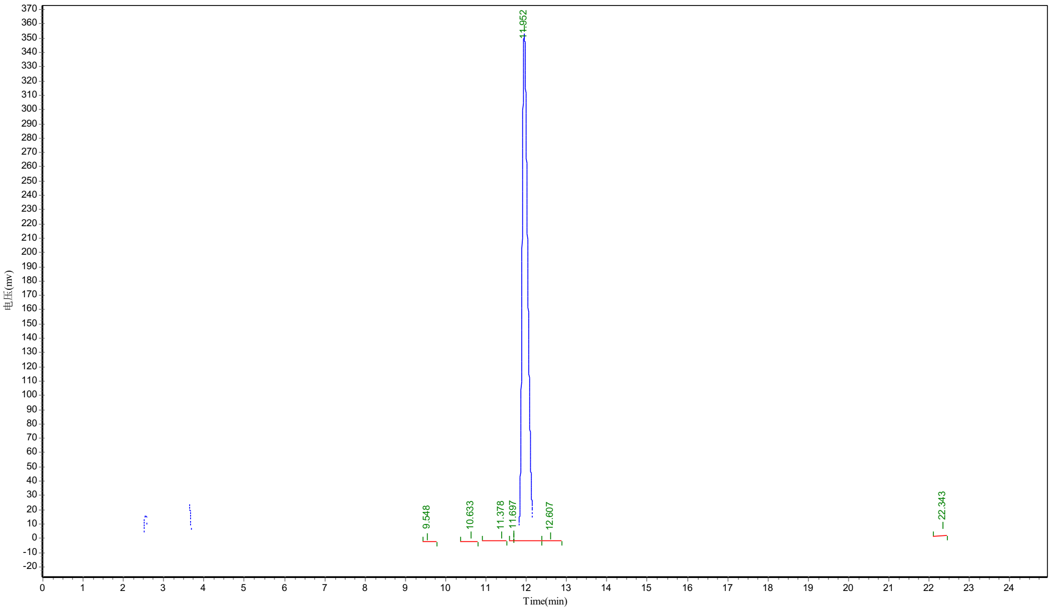


| Peak No. | Ret Time | Height | Area | Conc. |
|---|---|---|---|---|
| 1 | 9.548 | 501.438 | 4447.403 | 0.1253 |
| 2 | 10.633 | 1549.098 | 19702.748 | 0.5553 |
| 3 | 11.378 | 1028.513 | 14110.201 | 0.3976 |
| 4 | 11.697 | 1630.753 | 3942.228 | 0.1111 |
| 5 | 11.952 | 355993.063 | 3461311.750 | 97.5445 |
| 6 | 12.607 | 974.325 | 11727.622 | 0.3305 |
| 7 | 22.343 | 4396.594 | 33201.152 | 0.9357 |
| Total | | | | 100.00 |

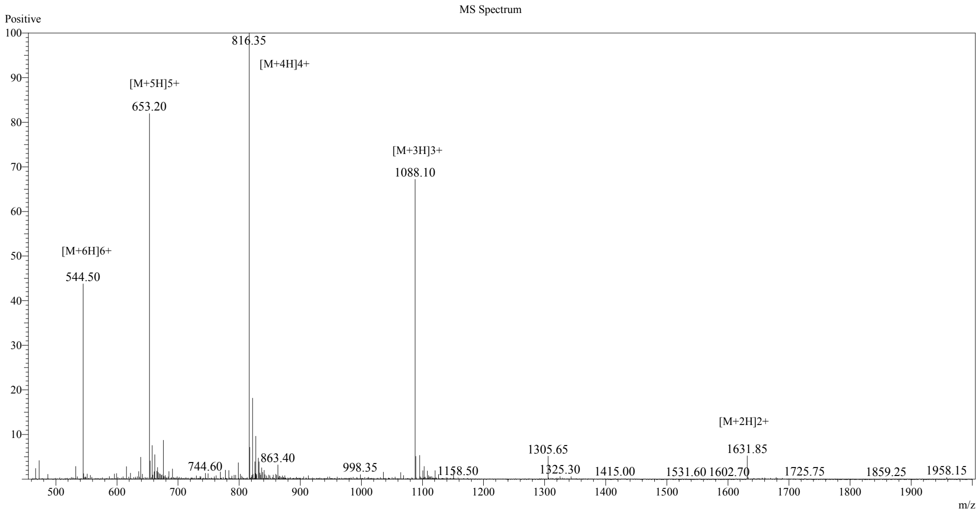


**Fig S13.** HPLC and mass-spectrometry of SP1 homologous peptide from *Candida albicans*.

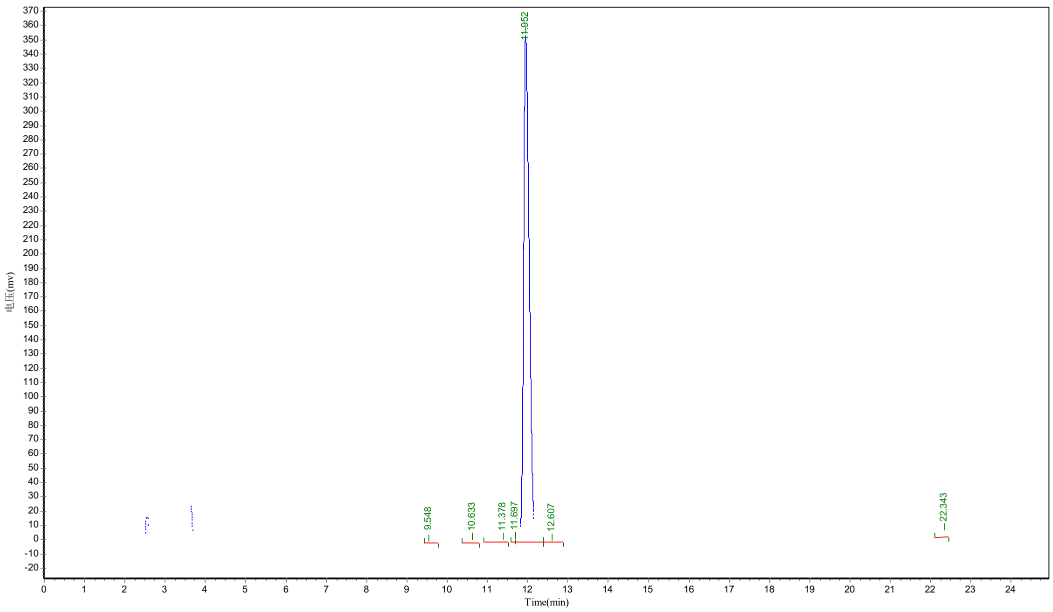


| Peak No. | Ret Time | Height | Area | Conc. |
|---|---|---|---|---|
| 1 | 9.548 | 501.438 | 4447.403 | 0.1253 |
| 2 | 10.633 | 1549.098 | 19702.748 | 0.5553 |
| 3 | 11.378 | 1028.513 | 14110.201 | 0.3976 |
| 4 | 11.697 | 1630.753 | 3942.228 | 0.1111 |
| 5 | 11.952 | 355993.063 | 3461311.750 | 97.5445 |
| 6 | 12.607 | 974.325 | 11727.622 | 0.3305 |
| 7 | 22.343 | 4396.594 | 33201.152 | 0.9357 |
| Total | | | | 100.00 |

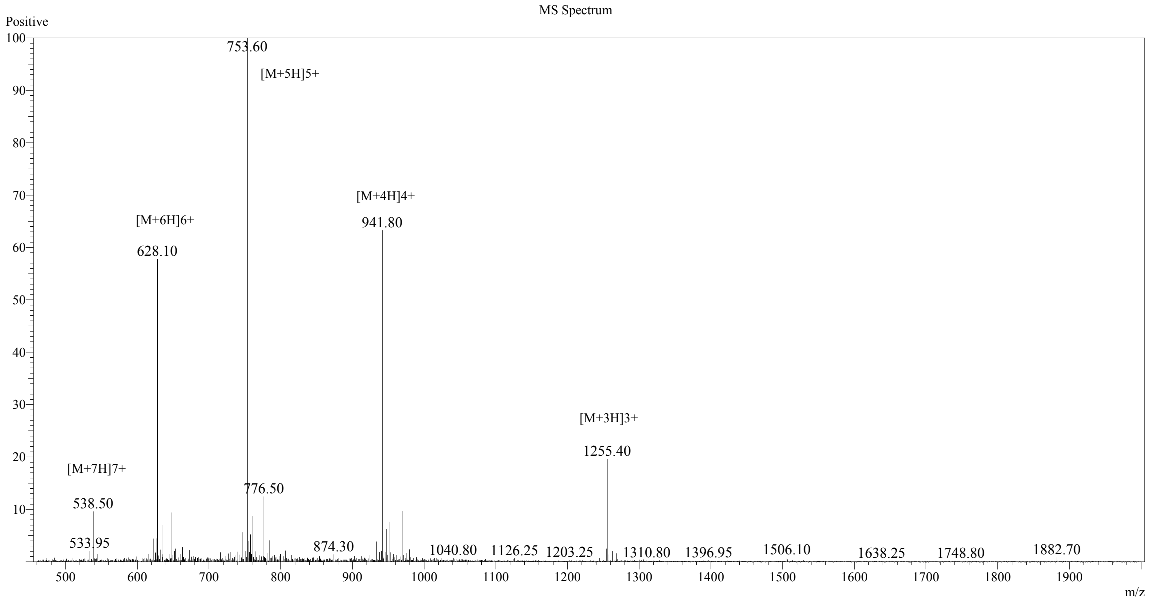


**Fig S14**. HPLC and mass-spectrometry of SP1 homolougus peptide from *Aspergillus fumigatus*.

**Table S1. The MICs of SP1 against different bacteria** (The MIC was determined from three independent experiments performed in triplicate).

| Strain | MIC(μM) |
|---|---|
| *Pseudomonas aeruginosa* | >128 |
| *Escherichia coli* | >128 |
| *Micrococcus luteus* | >128 |
| *Staphylococcus aureus* | >128 |

**Table S2. *Cryptococcus neoformans* or *Cryptococcus gattii* strains used in this study**

| Strain | Variety | Molecular type |
|---|---|---|
| H99 | *grubii* | VNI |
| JEC21 | *neoformans* | VNIV |
| W82 | *grubii* | VNI |
| W83 | *grubii* | VNII |
| W1010 | *gattii* | VGII |
| W1011 | *gattii* | VGI |
| R272 | *gattii* | VGIIb |
| WM179 | *gattii* | VGI |
| W2520 | *gattii* | VGIII |
| W1012 | *gattii* | VGIII |

**Table S3. MIC values of homologous peptides from different species to *C.neoformans* H99**

| Species | MIC (μM) |
|---|---|
| *Cryptococcus neoformans* | >128 |
| *Candida albicans* | >128 |
| *Aspergillus fumigatus* | >128 |
